# Integrating teamwork, clinician occupational well-being and patient safety – development of a conceptual framework based on a systematic review

**DOI:** 10.1186/s12913-016-1535-y

**Published:** 2016-07-19

**Authors:** Annalena Welp, Tanja Manser

**Affiliations:** Industrial Psychology and Human Factors, Department of Psychology, University of Fribourg, Rue Faucigny 2, 1700 Fribourg, Switzerland; Institute for Patient Safety, University Hospital Bonn, Stiftsplatz 12, 53111 Bonn, Germany; Department of Management, Technology & Economics, ETH Zurich, Weinbergstrasse 56/58, 8092 Zurich, Switzerland

**Keywords:** Teamwork, Clinician well-being, Patient safety, Framework, Systematic review

## Abstract

**Background:**

There is growing evidence that teamwork in hospitals is related to both patient outcomes and clinician occupational well-being. Furthermore, clinician well-being is associated with patient safety. Despite considerable research activity, few studies include all three concepts, and their interrelations have not yet been investigated systematically. To advance our understanding of these potentially complex interrelations we propose an integrative framework taking into account current evidence and research gaps identified in a systematic review.

**Methods:**

We conducted a literature search in six major databases (Medline, PsycArticles, PsycInfo, Psyndex, ScienceDirect, and Web of Knowledge). Inclusion criteria were: peer reviewed papers published between January 2000 and June 2015 investigating a statistical relationship between at least two of the three concepts; teamwork, patient safety, and clinician occupational well-being in hospital settings, including practicing nurses and physicians. We assessed methodological quality using a standardized rating system and qualitatively appraised and extracted relevant data, such as instruments, analyses and outcomes.

**Results:**

The 98 studies included in this review were highly diverse regarding quality, methodology and outcomes. We found support for the existence of independent associations between teamwork, clinician occupational well-being and patient safety. However, we identified several conceptual and methodological limitations. The main barrier to advancing our understanding of the causal relationships between teamwork, clinician well-being and patient safety is the lack of an integrative, theory-based, and methodologically thorough approach investigating the three concepts simultaneously and longitudinally. Based on psychological theory and our findings, we developed an integrative framework that addresses these limitations and proposes mechanisms by which these concepts might be linked.

**Conclusion:**

Knowledge about the mechanisms underlying the relationships between these concepts helps to identify avenues for future research, aimed at benefiting clinicians and patients by using the synergies between teamwork, clinician occupational well-being and patient safety.

**Electronic supplementary material:**

The online version of this article (doi:10.1186/s12913-016-1535-y) contains supplementary material, which is available to authorized users.

## Background

Patient safety is an important indicator of hospitals’ organizational performance. Approximately 10 % of patients suffer adverse events and half of those are deemed preventable [1]. Vincent defined patient safety as the absence of preventable adverse events – events that are a consequence of healthcare interventions rather than the patients’ condition [2]. Healthcare is predominantly provided by teams – two or more people each with specialized roles and responsibilities whilst interacting with the shared goal of patient care [3]. Consequently, in addition to medical competence, effective teamwork is critical for safe patient care [4–7]. This includes both observable team behaviors and clinicians’ perceptions of interpersonal team processes. For example, several studies have linked better coordination or team psychological safety to fewer medical errors and better patient outcomes such as length of stay [8–10]. Also, specific team *behaviors*, for example leadership, information sharing or decision making and team properties (e.g., shared mental models) are associated with performance indicators such as decision and execution latency or protocol adherence [5, 11, 12].

Teamwork is also an important predictor of another indicator of hospitals’ organizational performance: the well-being of healthcare providers [13, 14]. Reduced occupational well-being or high psychological strain may develop as an immediate or long-term response to stressors [15] and is highly prevalent in healthcare workers [16, 17]. Teamwork may constitute such a stressor. For instance, dysfunctional inter-professional teamwork predicts increased acute and chronic clinician strain [18, 19]. However, effective teamwork may protect team members from the effects of work stress, since positive perceptions of teamwork are associated with enhanced occupational well-being indicators such as better mental health in nurses and physicians [20, 21].

Lastly, clinicians’ occupational well-being and patient safety are interrelated. Reduced clinician occupational well-being is associated with objective and subjective patient safety indicators such as mortality ratios, clinician-rated safety and reported errors [13, 22, 23]. Highly strained clinicians might thus pose a threat to patient safety since patient safety incidents are stressors that may lead to decreased clinician well-being: clinicians report increased emotional distress following medical error [24].

Studies investigating associations between teamwork, clinician occupational well-being and patient safety originate from very different strands of research – medical, nursing, and psychology. So far, the evidence generated has not been drawn together for systematic evaluation. While this research showed that relationships exist between the independent associations of teamwork, clinician occupational well-being and patient safety, few studies investigated them simultaneously. Moreover, the *mechanisms* underlying the relationships and causalities between either two – and potentially all three – concepts are largely unknown.

To overcome this research gap, we aimed to provide an overview of the current state of research on relationships between at least two of the three concepts of teamwork, clinician occupational well-being, and patient safety in hospital settings. In a systematic review, we summarized theoretical foundations, sample, methodology, and empirical findings, and evaluated overall study quality. Based on relevant psychological theories and on the findings of the systematic review, we developed a conceptual framework integrating the three concepts. Specifically, we propose theoretically informed causal relationships between the concepts, describe focal points of past research, and identify gaps in the current knowledge. The framework is intended to serve as a blueprint both for future studies intended to benefit clinicians’ occupational well-being and patients’ safety.

## Methods

### Definition of central concepts

#### Teams and teamwork

In order to include a diverse array of healthcare teams, we used rather broad definitions of teams and teamwork. A team is defined as a group of two or more people embedded in an organizational system with specialized roles who are interdependent and socially interact with each other in order to reach a common goal [3]. Studies were included if the teams investigated matched these criteria. We based our definition of teamwork on the model by Marks and colleagues, which includes transition (planning, goal formulation), action (coordination, monitoring), and interpersonal processes (conflict management, motivation, or team members’ perceptions thereof, e.g., team climate) [25]. Thus, we excluded studies comparing the effects of team-based work to other forms of work organization. We included leadership if it was clearly directed at the team level, and excluded studies examining dyadic or organizational leadership processes. Lastly, we excluded studies assessing inter-team processes, because we were interested in how working *within* a team relates to patient safety and clinician well-being.

#### Clinician occupational well-being

Under occupational well-being, our aim was to identify studies investigating both positive and negative aspects [26–28]. We specifically included studies, based on Lazarus’ stress model, which investigated work-related psychological or physiological strain as an individual’s short- or long-term perception of, or response to, stressors at work, such as burnout [15]. In the case of workplace stressors, these are often referred to as job demands. According to the job demands-resources model, job demands are defined as physical, social, or organizational job characteristics that require increased effort, thereby depleting the individual’s energy and eventually decreasing occupational well-being or increasing strain [29]. We included studies examining mental fatigue (i.e., exhaustion or lack of energy that is not due to physical overexertion) if direct measures of mental fatigue were used rather than being inferred from external indicators such as shift duration [30]. Furthermore, we included general or work-related positive indicators of occupational well-being as an outcome of lack of job demands, or the abundance of job resources, such as work engagement. Job resources are physical, social, or organizational characteristics that help maintain the individual’s energy, thereby increasing occupational well-being or reducing the strain caused by job demands [29]. Our aim was to focus the review on studies examining occupational well-being as the result of appraisal of a stressor or lack thereof. For this reason, we excluded studies examining aspects of occupational well-being in the wider sense, i.e., studies investigating aspects that are the result of a large array of workplace characteristics, such as job satisfaction or organizational commitment. We furthermore excluded studies examining personality traits or psychopathological disorders. Lastly, we excluded long-term chronic somatic disorders such as lower back pain, as it is often unclear whether these conditions are caused by continuous psychological strain or physical activities.

#### Patient safety

We defined patient safety as “the avoidance, prevention, and amelioration of adverse outcomes or injuries stemming from the process of healthcare” [31]. We included studies covering variables that could directly affect a patient’s health status (i.e., reported or observed errors, key actions not being performed), as well as subjective patient safety ratings and objective morbidity-mortality-data. We excluded studies assessing quality of patient care or using safety climate as a substitute outcome measure.

### Search strategy

We searched six databases (Medline, PsycArticles, PsycInfo, Psyndex, ScienceDirect, and Web of Knowledge) to identify relevant literature. Our *a priori* assumption was that teamwork, clinicians’ occupational well-being and patient safety are related to each other. Thus, we combined two of the three keywords TEAMWORK, PATIENT SAFETY, WELL-BEING with AND. We then combined the results with OR. In order to receive both relevant and manageable results, we applied a number of strategies (e.g., MeSH/thesaurus terms, related terms, alternative spellings, truncations or plural forms, and adjacency terms; the complete search strategy for one database can be viewed in Additional file 1). Further inclusion criteria were: peer-reviewed journal articles, published in English between January 2000 and June 2015, referring to a hospital setting. We included studies sampling practicing nurses or physicians. If multiple publications were based on the same dataset, we either selected the paper that was first published or reported the most extensive data analysis. Finally, we hand-searched reference lists of the selected articles and systematic reviews we identified in our initial search.

### Screening and selection procedure

Two raters screened (AW and either MD, SS, or JV) all references independently. We scanned the title and abstract at the first stage and included studies investigating at least two of the three concepts (teamwork, patient safety, clinician well-being) in a hospital setting. At the second stage, we included studies reporting a statistical relationship between at least two of the relevant concepts, which clearly described measurement methods and were published in peer-reviewed journals. Disagreements between raters at the first screening stage led to inclusion, after which we resolved disagreements at the second stage by consensus discussion.

### Quality rating

To systematically assess study quality, we combined and slightly adapted existing systems. [32, 33] Ratings were based on a maximum of 19 items (not all items were applicable for all studies) covering topics such as validity of measures or statistical analyses. Items were rated as 0 = major limitations/not applicable/not mentioned, 0.5 = some limitations, or 1 = fulfilled. Two raters (AW and MD) independently evaluated study quality and resolved disagreements through discussion. All quality rating items are available in Additional file 2.

### Data extraction

We extracted study setting, study design, method of data collection, data analysis, and study outcomes from the selected studies. If results were described in sufficient detail but effect sizes were not reported, we calculated them according to convention [34, 35] to give an indication of whether a statistically significant relationship was large enough to infer practical significance (see Table 1 for an overview of effect size magnitudes) [36]. In some studies, teamwork, clinician occupational well-being and patient safety may have been analyzed within a larger context (e.g., nurse working environment), however, only relationships between the variables of interest to this review are reported.

**Table 1:** Overview of effect sizes [34, 35, 147]

Effect size	Abbreviation	Small	Medium	Large
Coefficient of determination	R^2^	.02	.13	.26
Cohen’s ƒ	ƒ	.14	.39	.59
Eta squared	η^2^	.01	.06	.14
Odds ratio	OR	1.5	3.5	7.0
Pearson correlation	r	.10	.30	.50

### Framework development

Building on the results of our systematic review, the framework development followed two stages. Based on the assumption that teamwork, clinicians’ occupational well-being and patient safety are correlated, our aim was to provide a framework summarizing the current state of research and exploring the underlying mechanisms and causal directions between the concepts. First, we examined measures, samples, and definitions of teamwork, well-being and patient safety to provide an overview of the evidence, and to detect trends and shortcomings in current research. Second, we drew from the theoretical foundations of the reviewed studies and from psychological theories relevant to the topic to aid interpretation of the findings and formulate hypotheses regarding the *causal* relationships between teamwork, clinician occupational well-being and patient safety to point out avenues for future research.

## Results

**Fig. 1:**
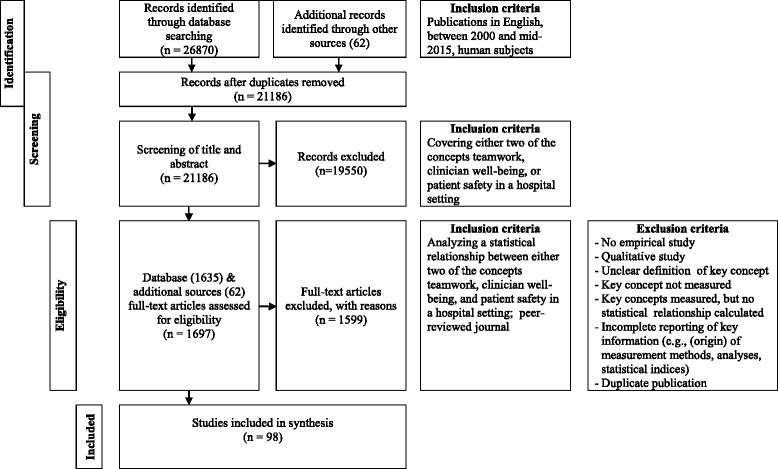
Flow diagram illustrating search method and inclusion/exclusion criteria

The database search from January 2000 to June 2015 yielded 26,870 results. We identified an additional 62 publications through other sources (e.g., hand-searching references lists). After removing duplicates, 21,186 publications remained. Following title and abstract screening, we retrieved the full text of 1697 publications. Examining full-texts and hand-searching reference lists led to the inclusion of 98 publications (see Fig. 1). Of these, 25 (26 %) investigated relationships between teamwork and well-being, 43 (44 %) between teamwork and patient safety, 25 (26 %) between well-being and patient safety, and five (5 %) included all three concepts.

### Quality rating

Selected studies were of medium (49 studies) or high quality (49 studies; see Tables 2, 3, 4 and 5 for individual quality scores). Average study quality was similar across the three concepts; teamwork, well-being and patient safety (i.e., 11.48 for teamwork/well-being [*SD* = 1.68], 11.03 for teamwork/patient safety [*SD* = 2.04], 10.92 for well-being/patient safety [*SD* = 2.013], and 11.20 [*SD* = 0.75] for teamwork/well-being/patient safety)). We excluded the low quality studies identified in this review at an early stage because the methodological description was insufficient for data extraction and assessment of quality (see Fig. 1).

**Table 2:** Relationships between teamwork and well-being

Study	Topic	Primary topic	Sample & setting	Design & data collection methods	Assessment of variables	Analyses	Findings	Outcomes & effect sizes	Quality score^d^
Bobbio et al., 2012 [38]	Mediation of relationship between empowering leadership/organizational support and burnout by trust in leader/organization	no	273 nurses, general hospital, Italy	Cross-sectional self-report questionnaire	Team leadership: Empowering leadership scale^a^ Well-being: Maslach Burnout Inventory (MBI)^a^	Path analysis	1) Satisfactory model fit 2) Trust in leader mediates relationship leading by example and emotional exhaustion 3) Trust in leader mediates relationship between showing concern/ interacting with the team and a) emotional exhaustion b) cynicism 4) Trust in organization mediates relationship between informing and a) emotional exhaustion and b) cynicism 5) No mediation effects for reduced professional efficacy	1) *χ* ^2^ (18) = 21.27, *p* = 0*.*27, *χ* ^2^/df = 1*.*18, RMSEA = 0*.*03, CFI = 1*.*00, SRMR = 0*.*02 Indirect effects: 2) β = −0*.*04, *p* < 0*.*05 3a) β = −0*.*23, *p* < 0*.*001 3b) β = −0*.*15, *p* < 0*.*001 4a) β = −0*.*03, *p* < 0*.*05 4b) β = −0*.*04, *p* < 0*.*02 5) NS	11.5 (16)
Bratt et al., 2000 [39]	Relationshi*p*s between nurse/unit characteristics, work environment and job satisfaction	no	1973 nurses, 70 pediatric intensive care units, 65 pediatric hospitals, USA/Canada	Cross-sectional self-report questionnaire	Teamwork: a) Group cohesion: Group Judgment Scale b) Nurse-physician collaboration: Collaboration and Satisfaction about Care Decisions^a^ Well-being: Job Stress Scale^a^	Pearson’s correlation	Job stress is negatively correlated with 1) group cohesion 2) nurse-physician collaboration	1) *r* = −0*.*43, *p* < 0*.*001 2) *r* = −0*.*37, *p* < 0*.*001	9*.*5 (16)
Brunetto et al., 2011 [40]	Relationships between supervisor-subordinate relationship, teamwork, role ambiguity and well-being	yes	1138 nurses, 3 public and 7 private urban and regional hospitals, Australia	Cross-sectional self-report questionnaire	Teamwork: Nurses’ Satisfaction with Teamwork Scale Well-being: Perception of Well-being Scale (self-developed)	Pearson’s correlation	Positive correlation between nurses’ satisfaction with teamwork and well-being	Public sector: *r* = 0*.*35, *p* < 0*.*001 Private sector: *r* = 0*.*39, *p* < 0*.*001	9*.*5 (16)
Brunetto et al., 2013 [48]	Workplace relationships, engagement, well-being, commitment, and turnover	no	1228 nurses, Australia / USA	Cross-sectional self-report questionnaire	Teamwork: Satisfaction with teamwork^a^ Well-being: employee engagement^a^, well-being scale developed by first author	Structural equation modeling (SEM)	Teamwork is positively associated with 1) engagement and 2) well-being in the a) Australian and b) US sample	1a) B = .19, *p* < .001 1b) B = .24, *p* < .001 2a) β = .30, *p* < .001 2b) β = .37, *p* < .001	12 (16)
Bruyneel et al., 2009 [41]	Relationship between nurse working environment and nurse-perceived outcomes	no	179 nurses, 12 units, 5 acute care hospitals, Belgium	Cross-sectional self-report questionnaire	Teamwork: Nursing Work Index-Revised (NWI-R)^a^ subscale *Nurse-Physician-Relations* Well-being: MBI^a^	Multivariate logistic regression	Nurse-physician relations are not associated with emotional exhaustion	NS	11*.*5 (16)
Budge et al., 2003 [20]	Relationships between nurses’ work characteristics, work relationships and health	no	225 nurses, general hospitals, New Zealand	Cross-sectional self-report questionnaire	Teamwork: Nurse-Physician-Relations Scale^a^ Well-being: Short-Form Health Survey (SF-36)^a^ subscales *mental health* and *vitality*	Pearson’s correlation	Positive correlation between nurse-physician relations and 1) mental health 2) vitality	1) *r* = 0*.*29, *p* < 0*.*001 2) *r* = 0*.*36, *p* < 0*.*001	12*.*5 (16)
Cheng et al., 2013 [49]	Relationships between team climate, emotional labor, burnout, quality of care, and turnover	no	201 nurses, 1 hospital, Australia	Cross-sectional self-report questionnaire	Teamwork: Team Climate Inventory (TCI)^a^ Well-being: Oldenburg Burnout Inventory (OLBI)^a^	Structural equation modeling	1) Good overall model fit 2) Team climate is negatively associated with burnout	1) *χ* ^2^ = 241.31; *χ* ^2^/df = 11.49; TLI = .95; CFI = .98; RMSEA = .051 2) β = −.37, *p* < .01	13 (16)
Gabriel et al., 2011 [18]	Collegial nurse-physician relations and psychological resilience moderate relationships between task accomplishment satisfaction and pre-/postshift affect	no	57 nurses, 1 hospital, USA	Cross-sectional pen-and-paper diary-report	Teamwork: Nurse-Physician-Relations Scale^a^ Well-being: Affect scale, psychological resilience based upon Connor–Davidson Resilience Scale (CD-RISC)^a^	Pearson’s correlation, multilevel modeling	1) Nurse-physician relations are a) negatively correlated with preshift negative affect b) positively correlated with preshift positive affect 2) No correlations between nurse-physician-relations and psychological resilience 3) Nurse-physician relations a) negatively predict postshift negative affect b) positively predict postshift positive affect	1a) *r* = 0*.*30, *p* < 0*.*05 1b) *r* = 0*.*33, *p* < 0*.*05 2) NS 3a) γ = −0*.*13, *p* < 0*.*01 3b) γ = 0*.*2, *p* < 0*.*01	12 (16)
Gevers et al., 2010 [54]	Relationship between acute/chronic job demands and acute job strain and relationship between the latter and individual teamwork behavior	yes	48 nurses, nursing students and physicians, emergency department, The Netherlands	Cross-sectional self-report questionnaire	Teamwork and well-being: self-developed items adapted from existing measures	(Hierarchical) linear regression	1) Acute a) cognitive strains b) emotional strains separately negatively predict individual teamwork behavior c) whereas physical strains do not 2) When all three predictors are analyzed simultaneously, only acute emotional strains remain significant	1a) β = −0*.*35, *p* < 0*.*01, *R* ^*2*^ = 0*.*18, [*f* ^*2*^ = 0*.*22]^b,c^ 1b) β = −0*.*44, *p* < 0*.*001, *R* ^*2*^ = 0*.*25, [*f* ^*2*^ = 0*.*33]^b,c^ 1c) NS 2) β = −0*.*36, *p* < 0*.*05, *R* ^*2*^ = 0*.*26, [*f* ^*2*^ = 0*.*35]^b,c^, emotional & physical strains: NS	13 (16)
Gunnarsdottir et al., 2009 [42]	Relationships between nurses’ work environment and work outcomes	no	695 nurses, various specialties, university hospital, Iceland	Cross-sectional self-report questionnaire	Teamwork: Nurse-Physician-Relations Scale^a^ Well-being: Emotional Exhaustion^a^	(Hierarchical) linear regression	1) Nurse-physician relations are negatively associated with emotional exhaustion 2) Upon inclusion of four additional predictors, this association becomes non-significant	1*)* β = −2*.*38, *p* < 0*.*001, []^b^ 2) NS	12*.*5 (16)
Kanai-Pak et al., 2008 [43]	Relationships between nurses’ work environment and work outcomes	yes	5956 nurses, various specialties, 19 hospitals, Japan	Cross-sectional self-report questionnaire	Teamwork: Nurse-Physician-Relations Scale^a^ Well-being: Emotional Exhaustion^a^	Multivariate logistic regression	Lower nurse-physician relations are associated with higher risk for emotional exhaustion	Adj. OR = 1*.*35, *p* < 0*.*05	10*.*5 (16)
Klopper et al., 2012 [44]	Relationships between nurses’ work environment, job satisfaction and burnout	no	935 nurses, ICU, 62 hospitals, South Africa	Cross-sectional self-report questionnaire	Teamwork: Nurse-Physician-Relations Scale^a^ Well-being: MBI^a^	Spearman’s rank correlation	1) Negative correlation between nurse–physician relations and a) emotional exhaustion b) depersonalization 2) Positive correlation between nurse–physician relations and personal accomplishment	1a) ρ = −0*.*255, *p* < 0*.*01 1b) ρ = −0*.*193, *p* < 0*.*01 2) ρ = 0*.*199, *p* < 0*.*01	8*.*5 (16)
Lehmann-Willenbrock et al., 2012 [45]	Mediation of relationships between appreciation of age diversity and nurse Well-being/team commitment by co-worker trust	yes	138 nurses, 1 hospital, Germany	Cross-sectional self-report questionnaire	Teamwork: Team commitment scale Well-being: Workplace Irritation Scale^a^	Pearson’s correlation	Negative correlation between team commitment and irritation	*r* = −0*.*33, *p* < 0*.*01	12*.*5 (16)
Li et al., 2013 [50]	Relationships between nurse work environment and burnout	no	23 446 nurses, 2087 units, 352 hospitals, 11 European countries	Cross-sectional self-report questionnaire	Teamwork: Nurse-physician relations^a^ Well-being: MBI^a^	Multilevel regression	1) As expected, nurse-physician relations on the a) unit, but not on the b) hospital or c) country level are negatively related to emotional exhaustion on the individual level 2) As expected, nurse-physician relations on the a) unit, but not on the b) hospital or c) country level are negatively related to depersonalization on the individual level 3) As expected, nurse-physician relations on the a) unit, but not on the b) hospital or c) country level are positively related to personal accomplishment on the individual level	1a) B = −0.11; 95 % equal tail credibility interval (ETCI) -0.21 to −0.002 1b) NS 1c) NS 2a) B = −0.17; 95 % ETCI −0.27 to -.07 2b) NS 2c) NS 3a) B = 0.20; 95 % ETCI −0.29 to -.12 3b) NS 3c) NS	13.5 (16)
Pisarski & Barbout, 2014 [37]	Relationships between team climate, roster control, work-life conflict and fatigue	yes	166 nurses, 1 hospital, Australia	Longitudinal self-report questionnaire	Teamwork: 10 items adapted from teamwork climate measure developed by authors Well-being: 2 items from Standard Shiftwork Index (SSI)	Multiple hierarchical regression	1) Overall, team climate at time 1 does not predict fatigue at time 2 2) Team climate of day shift nurses is negatively related to fatigue	1) NS 2) β = −.16, *p* < .05	13 (16)
Profit et al., 2013 [57]	Relationships between burnout and patient safety culture	yes	2073 nurses and other healthcare professionals in 44 neonatal intensive care unit	Cross-sectional self-report questionnaire	Teamwork: Safety Attitudes Questionnaire (SAQ)^a^ subscale *teamwork climate* Well-being: 4-item version of MBI^a^	Pearson correlation	Negative correlation between burnout and teamwork climate	*r* = −.38, *p* < .05	11 (16)
Rafferty et al., 2001 [46]	Relationship between interdisciplinary teamwork and nurse autonomy on patient and nurse outcomes and nurse assessed quality of care	yes	5006 nurses, 32 hospitals, UK	Cross sectional self-report questionnaire	Teamwork: Items referring to teamwork on unit derived from NWI-R^a^ Well-being: MBI^a^	Pearson’s correlation	Negative correlation between teamwork and burnout	*r* = −0*.*219, *p* < 0*.*001	6*.*5 (16)
Raftopoulos et al., 2011 [53]	Relationships between safety and teamwork climate and stress	no	106 midwives, public maternity units, Cyprus	Cross-sectional self-report questionnaire	Teamwork: Safety Attitudes Questionnaire (SAQ)^a^ subscale *teamwork climate* Well-being: job exhaustion, occupational stress (1 item each)	Backward stepwise linear regression	1) Job exhaustion negatively predicts teamwork climate (14 predictors altogether) 2) No association between teamwork and occupational stress	1) β = −12*.*85, *p* = 0*.*046, *R* ^*2*^ = 0*.*117, [*f* ^*2*^ = 0*.*13]^b,c^ 2) NS	10 (16)
Rathert et al., 2012 [55]	Mediation of relationship between nurses’ work environment and workarounds by emotional exhaustion	no	272 nurses & other medical care providers, acute care hospital, North America	Cross-sectional self-report questionnaire	Teamwork: 4 items from Agency for Healthcare Research and Quality (AHRQ) Patient Safety Culture Survey^a^ Well-being: Emotional Exhaustion^a^	Path analysis	1) Negative association between teamwork and emotional exhaustion within larger path model 2) Good final model fit	1) β = −0*.*19, *p* < 0*.*01 2) GFI = 0*.*99, AGFI = 0*.*92, NNFI = 0*.*97, RMSEA = 0*.*06, *χ* ^2^ = 11*.*81 (*df* = 6)	11*.*5 (16)
So et al., 2011 [56]	Cultural differences in relationships between team structure, job design, and Well-being	yes	470 nurses & other medical care providers, acute hospitals, China & UK	Cross-sectional self-report questionnaire	Teamwork: items about team structure (roles, objectives, cooperation, performance reflection) Well-being: items about perceived work stress	Path analysis	Negative association between team structure and work stress within larger path model 1) in the UK sample 2) but not in the Chinese sample 3) Good overall model fit	1) β = −0*.*18, *p* < 0*.*05, *R* ^*2*^_all stress predictors_ = 0*.*302 2) NS 3) *χ* ^2^ = 787*.*94 (*df* = 246, *p* = 0*.*05), CFI = 0*.*91, NNFI = 0*.*91, RMSEA = 0*.*071, 90 % CI 0*.*065 – 0*.*076	12*.*5 (16)
Sutinen et al., 2005 [21]	Relationships between health, work and social characteristics and retirement attitudes	no	447 physicians, several hospitals, Finland	Cross-sectional self-report questionnaire	Teamwork: TCI^a^ Well-being: General Health Questionnaire (GHQ-12)^a^	Pearson’s correlation	Negative correlation between teamwork and minor psychiatric morbidity	*r* = −0*.*12, *p* < 0*.*05	10*.*5 (16)
Van Bogaert et al., 2009 [47]	Mediation of relationships between nurse work environment and nurse job outcomes and quality of care by burnout	no	401 nurses, medical, 31 units, general and university hospital, Belgium	Cross-sectional self-report questionnaire	Teamwork: Nurse-Physician-Relations Scale^a^ Well-being: MBI^a^	Pearson’s correlation Path analysis	1) Negative correlation between nurse-physician relationship and a) depersonalization b) personal accomplishment 2) Within path model: negative association between nurse-physician relationship and emotional exhaustion 3) Adequate overall model fit	1a) *r* = 0*.*155, *p* < 0*.*05 1b) *r* = −0*.*115, *p* < 0*.*01 2) β = −0*.*19 3) *χ* ^2^ = 548*.*1, *df* = 313, *p* < 0*.*001, CFI = 0*.*906, IFI = 0*.*903, RMSEA = 0*.*43	11*.*5 (16)
Van Bogaert et al., 2010 [19]	Relationships between nurse work environment, nurse job outcomes, quality of care, and burnout	no	546 nurses, 42 units, general and university hospitals, Belgium	Cross-sectional self-report questionnaire	Teamwork: Nurse-Physician-Relations Scale^a^ Well-being: MBI^a^	Linear mixed effects multilevel model	1) Positive association between nurse-physician relationship and personal accomplishment 2) Negative association between nurse-physician relationship and a) emotional exhaustion b) depersonalization	1) β = 1*.*98, *p* < 0*.*0001 2a) β = −3*.*79, *p* < 0*.*0001 2b) β = −1*.*09, *p* < 0*.*05	11*.*5 (16)
Van Bogaert et al., 2013 [52]	Relationships between nurse work environment, nurse characteristics, burnout, nurse job outcomes, and quality of care	no	1201 nurses, 116 units, 8 hospitals, Belgium	Cross-sectional self-report questionnaire	Teamwork: nurse-physician relations subscale of NWI^a^ Well-being: MBI^a^	Structural equation modelling (SEM)	1) Satisfactory overall model fit 2) No relationship between nurse-physician relations and emotional exhaustion 3) Negative correlation between nurse-physician relations and depersonalization but no relationship in final SEM 4) Positive correlation between nurse-physician relations and personal accomplishment but no relationship in final SEM	1) CFI = .90, IFI = .90, RMSEA = .43 2) NS 3) *r* = −.08, *p* < .01 4) *r* = .11, *p* < .01	13 (16)
Van Bogaert et al., 2014 [51]	Relationships between role-, job- and organizational characteristics, and occupational stress and well-being	no	365 nurse unit managers, Belgium	Cross-sectional self-report questionnaire	Teamwork: nurse-physician relations subscale of Leiden Quality of Work Questionnaire for Nurses (LQWQ-N)^a^ Well-being: emotional exhaustion subscale from MBI^a^; Utrecht Work Engagement Scale (UWES)^a^	Hierarchical multiple regression	1) Nurse-physician relations negatively predict emotional exhaustion 2) Nurse-physician relations do not predict work engagement	1) β = −.22, *p* < .01 2) NS	14 (16)

We report not only significant but also non-significant relationships between predictor and outcome variables of interest in this review as hypothesized in the reviewed studies; even if not explicitly stated in the original publication

^a^validated instrument

^b^effect sizes calculated by authors, calculation not possible if brackets empty

^c^Cohen’s*ƒ* ^2^ based on R^2^ instead of ΔR^2^

^d^in brackets: maximal possible score

**Table 3:** Relationships between teamwork and patient safety

Study	Topic	Primary topic	Sample & setting	Design & data collection methods	Assessment of variables	Analyses	Findings	Outcomes & effect sizes	Quality score^d^
a) observational studies									
Burtscher et al., 2010 [61]	Relationships between coordination activities and team performance under differing situational demands	yes	19 anesthetists and 14 anesthesia nurses, 40 cases, teaching hospital, Switzerland	Video observation of anesthesia induction	Teamwork: observation system used for coding coordination activities & clinical work Patient safety: team performance (self-developed checklist)	Paired-sample *t*-test	1) Compared to low-performing teams, high-performing teams increase task management during non-routine events 2) No changes in information management during non-routine events	1) *t*(20) = −2*.*75, *p* < 0*.*05, []^b^ 2) NS	13*.*5 (15)
Burtscher et al., 2011 [12]	Relationships between adaptive team coordination during non-routine events and clinical performance during anesthesia induction	yes	15 anesthesia teams (1 resident, 1 nurse), teaching hospital, Switzerland	Video observation of simulated anesthesia induction	Teamwork: team coordination (structured observation)^a^ Patient safety: decisions and execution latency (expert rating)	Pearson’s correlation	1) Information management is a) negatively correlated with decision latency b) but not with execution latency 2) No correlations between task management and a) decision latency b) execution latency	1a) *r* = −0*.*49, *p* = 0*.*003 1b) NS 2a) NS 2b) NS	12*.*5 (15)
Burtscher et al., 2011 [5]	Team mental model properties moderate link between monitoring behaviors and performance in anesthesia induction	yes	31 teams (1 anesthesia resident, 1 anesthesia nurse), teaching hospital, Switzerland	Video observation of simulated anesthesia induction	Teamwork: Team mental model similarity and accuracy (concept mapping), monitoring behavior (structured observation^a^) Patient safety: adherence to anesthesia induction protocol (structured observation^a^)	Multiple hierarchical regression	1) Teams with similar mental models perform well irrespective of team monitoring level; teams with dissimilar mental models only perform well when team monitoring is low 2) Team mental model similarity is only related to performance when team mental model accuracy is also high 3) Team performance is high when either team or system monitoring is high and the other is low 4) Mental model accuracy does not moderate relationship between systems monitoring and performance	1) β = 0*.*36, *p* = 0.04, Δ*R* ^2^ = 0*.*13, [*ƒ* ^2^ = 0*.*21]^b^ 2) β = 0*.*42, *p* = 0.02, Δ*R* ^2^ = 0*.*17, [*ƒ* ^2^ = 0*.*12]^b^ 3) β = −0*.*36, *p* = 0.04, Δ*R* ^2^ = 0*.*12, [*ƒ* ^2^ = 0*.*28]^b^ 4) NS	14 (15)
Catchpole et al., 2007 [64]	Relationships between non-technical skills and adverse events in the OR	yes	42 operations (24 pediatric, 18 orthopedic), 2 hospitals, UK	Live & video observation	Teamwork: non-technical skills (NOTECHS^a^) Patient safety: Adverse events: minor problems, intraoperative performance, operating time	Multiple linear regression	Non-technical skills negatively predict 1) minor problems but not 2) intraoperative performance or 3) operating time	1) *B* = −3*.*3, *t* = −2.2, *p* = 0*.*035, []^b^ 2) NS 3) NS	8 (15)
Catchpole et al., 2008 [62]	Relationships between non-technical skills and errors in the OR	yes	54 surgeons, anesthetists, and nurses, 48 operations (26 laparoscopic cholecystectomies, 22 carotid endarterectomies), 1 hospital, UK	Live observation of operation	Teamwork: NOTECHS^a^ Patient safety: errors in surgical technique (observation clinical human reliability assessment technique), other procedural problems and errors (checklist), operating time	Multiple linear regression	1a) Surgical leadership and management negatively predicts operating time, 1b) whereas anesthetic leadership and management in carotid endarterectomy positively predicts operating time 2a) nursing leadership and management negatively predict other procedural problems and errors 2b) whereas nursing leadership and management in carotid endarterectomy positively predicts operating time (2 predictors) 3a) surgical situation awareness negatively predicts errors in surgical techniques (3 predictors) 3b) whereas surgical situation awareness in carotid endarterectomy positively predicts operating time (3 predictors) 4) Teamwork dimensions a) leadership and management b) teamwork and cooperation c) problem solving and decision making d) situation awareness are not associated with patient safety dimensions e) errors in surgical technique f) other procedural problems and error g) operating time	1a) β = −0*.*19, *p* = 0*.*023 1b) β = 0*.*81, *p* < 0.001, *R* ^2^ = 0.717, [*ƒ* ^2^ = 2*.*53]^b,c^ 2a) β = −0*.*39, *p* = 0*.*012 2b) β = 0*.*41, *p* = 0.008, *R* ^2^ = 0*.*69 [*ƒ* ^2^ = 2.215]^b,c^ 3a) β = −0*.*71, *p* < 0*.*001 3b) β = 1*.*97, *p* < 0.001, *R* ^2^ = 0.19, [*ƒ* ^2^ = 0*.*233]^b,c^ 4ae-dg) 9 non-significant associations	9 (15)
Catchpole et al., 2008 [63]	Relationships between non-technical skills and safety threats, errors, and operative duration	yes	Physicians and nurses, 44 operations (24 pediatric, 20 orthopedic), 2 hospitals, UK	Live & video observation	Teamwork: NOTECHS^a^ Patient safety: errors & threats (checklists and free observations)	Spearman’s rank correlation	1) Positive correlation between non-technical skills and 1a) safety threats 1b) operative duration 1c) but not technical errors in pediatric surgery 2) No correlations between non-technical skills and 1a) safety threats 1b) operating time 1c) technical errors in orthopedic surgery	1a) ρ = 0*.*58, *p* < 0*.*005 1b) ρ = 0*.*58, *p* < 0*.*005 1c) NS 2a) NS 2b) NS 2c) NS	10 (15)
Endacott et al., 2014 [81]	Relationships between leadership, teamwork and performance in medical emergencies	yes	42 nurses, 15 teams, 1 hospital, Australia	Video observation of simulated emergency	Teamwork: Team Emergency Assessment Measure (TEAM) ^a^ Patient safety: performance of key treatment actions	Pearson correlation	Teamwork correlates positively with patient safety in the 1) respiratory distress and 2) hypovolemic shock but not in the 3) chest pain scenario	1) *r* = .90, *p* < 0.001 2) *r* = .54, *p* < .05 3) NS	11.5 (15)
Kolbe et al., 2012 [65]	Relationships between speaking up and technical team performance/team interaction	no	31 anesthesia teams (1 nurse, 1 resident), teaching hospital, Switzerland	Video observation of simulated anesthesia induction	Teamwork: Coding scheme for (non-)verbal team interactions Patient safety: technical team performance (adherence to checklist of standard anesthesia induction and target values)	Hierarchical linear regression	1) Technical team performance is predicted by nurses’ levels of speaking up 2) but not by residents’ levels of speaking up	1*)* β = 0*.*43, *p* = 0.017, *R* ^*2*^ = 0*.*18, [*f* ^*2*^ = 0*.*22]^bc^ (2 predictors) 2) NS	14 (15)
Kuenzle et al., 2010 [67]	Relationship between shared leadership and anesthesia team performance under high and low task load	yes	12 anesthesia teams (1 resident, 1 nurse), teaching hospital, Switzerland	Video observation of simulated anesthesia induction	Teamwork: Coding scheme for content-oriented and structuring leadership behavior Patient safety: performance (reaction time after non-routine event)	ANOVA	1a) No differences in shared leadership behaviors of high-performing teams between nurses and residents 1b) during high- and low task load situations 2a) Residents show more leadership behaviors than nurses in low performing teams 2b) independent of task load	1a) *F*(1, 20) = 0.00, *p* = 0*.*971, η^2^ = 0*.*000 1b) Interaction: NS 2a) *F*(1, 20) = 7*.*14, *p* = 0*.*015, η^2^ = 0*.*263 2b) Interaction: NS	12*.*5 (15)
Kuenzle et al., 2010 [66]	Relationship between shared leadership and anesthesia team performance under high and low task load	yes	12 anesthesia teams (1 resident, 1 nurse), 1 hospital, Switzerland	Video observation of simulated anesthesia induction	Teamwork: structuring and content oriented leadership: structured observation Patient safety: performance (speed of correct management after non-routine event = high task load)	Spearman’s rank correlation Kruskal-Wallis-test	1) Under high task load team performance and a) structuring and b) content-oriented leadership are not correlated 2) Under low task load, team performance and a) structuring, b) but not content-oriented leadership are negatively correlated 3) Interaction of leadership behavior and team experience is not associated with team performance	1a) NS 1b) NS 2a) ρ = −0*.*56, *p* < 0*.*05 2b) NS 3) NS	12 (15)
Lubbert et al., 2009 [68]	Relationship between team organization and treatment errors	yes	378 video registrations of patients treated in the emergency room, 1 hospital, The Netherlands	Video observation	Teamwork & patient safety: Self-developed checklist measuring adherence to advanced trauma life support (ATLS) guidelines	*t*-test	1) Errors in team organization dimension *evident leadership* are associated with more deviations from treatment protocol, whereas 2) errors in team organization dimension *effective leadership* are not	1) *p* = 0*.*01 (no other indicators reported) 2) NS	6 (15)
Manser et al., 2009 [11]	Relationships between different coordination patterns and team performance	yes	46 anesthesia residents, 23 teams, USA	Video observation of simulated anesthesia emergency	Teamwork: self-developed coding scheme for coordination Patient safety: clinical performance (adherence to malignant hyperthermia treatment guidelines)	Hierarchical regression analysis	1) Time spent on coordination dimensions a) task management b) but not information management c) or coordination via work environment negatively predicts performance 2) Time spent on task management categories a) task distribution b) but not planning c) clarification d) initiating action e) or assistance negatively predicts performance 3) Time spent on information management categories a) situation assessment b) but not information transfer c) decision making d) or feedback/acknowledgement negatively predicts performance	1a) β = −0*.*47, *p* < 0.01, Δ*R* ^*2*^ = 0*.*243, [*f* ^*2*^ = 0*.*32]^b^ 1b) NS 1c) NS 2a) β = −0*.*54, *p* < 0.01, Δ*R* ^*2*^ = 0*.*340, [*f* ^*2*^ = 0*.*52]^b^ 2b) NS 2c) NS 2d) NS 2e) NS 3a) β = −0*.*57, *p* < 0.05, Δ*R* ^*2*^ = 0*.*227, [*f* ^*2*^ = 0*.*29]^b^ 3b) NS 3c) NS 3d) NS	11*.*5 (15)
McCulloch et al., 2009 [6]	Relationships between non-technical skills and technical errors	yes	54 surgeons, anesthetists and nurses, 48 observations before and 55 observations after training, teaching hospital, UK	Uncontrolled pre-post-training Live observations of operations	Teamwork: NOTECHS^a^ Patient safety: technical errors (Observation Clinical Human Reliability Assessment, OCHRA)^a^	Spearman’s rank correlation	1) Negative correlation between a) overall non-technical skills and technical errors b) especially for surgical sub-team 2) Negative correlation between a) situational awareness and technical errors b) especially for surgical sub-team	1a) ρ = −0*.*215, *p* = 0*.*024 1b) ρ = −0*.*236, *p* = 0*.*013 2a) ρ = −0*.*300, *p* = 0*.*001 2b) ρ = −0*.*436, *p* < 0*.*0001	11*.*5 (18)
Mishra et al., 2008 [69]	Relationships between non-technical skills and technical errors	yes	26 observations (nurses, surgeons, anesthetists), teaching hospital, UK	Live observation of operation	Teamwork: NOTECHS^a^ Patient safety: OCHRA^a^	Spearman’s rank correlation	1) No correlation between technical errors and a) leadership & management b) teamwork & cooperation c) problem-solving and decision-making in the d) overall team, or e) surgeon f) anesthetists g) and nurses subgroup 2) Negative correlation between situation awareness and technical errors for a) overall team b) surgeon subgroup c) but not anesthetists d) and nurses subgroup	1ad) NS 1ae) NS 1af) NS 1ag) NS 1bd) NS 1be) NS 1bf) NS 1bg) NS 1 cd) NS 1ce) NS 1cf) NS 1cg) NS 2a) ρ = −0*.*505, *p* = 0*.*009 2b) ρ = −0*.*718, *p* = 0*.*001 2c) NS 2d) NS	10 (15)
Ottestad et al., 2007 [70]	Development and psychometric testing of tool to measure resuscitative skills and to compare interns and teams regarding ideal management of septic shock	no	23 observations (ICU residents), USA	Video observation of emergency simulation	Teamwork: NOTECHS^a^ Patient safety: Adherence to Surviving Sepsis Campaign Guidelines	Pearson’s correlation	Positive correlation between non-technical skills and team sepsis management	*r* = 0*.*4, *p* = 0*.*05	7*.*5 (15)
Schmutz et al., 2015 [79]	Relationships between coordination, task type and performance in medical emergencies	yes	277 nurses, resident and senior physicians, 68 teams, 7 hospitals, Germany	Video observation of simulated pediatric emergency	Teamwork: Coordination behaviors via CoMeT–E (Coordination System for Medical Teams - Emergency) observation tool^a^ Patient safety: Clinical performance via key treatment steps checklist	Hierarchical linear regression	1a) Coordination behavior *closed-loop communication* is positively associated with clinical performance, whereas 1b) coordination behaviors *task distribution* and 1c) *providing information* without request are not. 2a) Task type moderates relationship 1a) in that it is stronger in rule-based compared to knowledge-based tasks 2b) Task type did not moderate relationship 1b) 2c) Task type did not moderate relationship 1c)	1a) β = .25, *p* < .05 1b) NS 1c) NS 2a) β = −.52, *p* < .01 2b) NS 2c) NS	14 (15)
Schraagen et al., 2011 [85]	Relationships between non-routine events, teamwork and patient outcomes	yes	1 pediatric cardiac surgery team, 40 operations, The Netherlands	Cross-sectional self-report questionnaire, live observation of operations, record review	Teamwork: observation tool derived from NOTECHS^a^, ANTS^a^, NOTSS^a^, and OTAS^a^ Patient safety: 30-day postsurgical complications, operating time	Pearson’s correlation, ANOVA	1) Positive correlation between non-technical skills and a) operating time b) but not postsurgical complications 2) Explicit coordination of anesthetists is associated with higher levels of postsurgical complications	1a) *r* = 0*.*45, *p* < 0*.*05 1b) NS 2) *M*_uncomplicated_ = 12*.*88, *M*_minor complications_ = 21*.*55, []^b^ *M*_major complications_ = 16*.*40, *F*(2,36) = 4*.*78, *p* < 0*.*01, []^b^	10 (16)
Schraagen et al., 2011 [86]	Relationships between non-routine events, teamwork and patient outcomes	yes	1 pediatric cardiac surgery team, 40 operations, The Netherlands	Cross-sectional self-report questionnaire, live observation of operations, record review	Teamwork: NOTECHS^a^ Patient safety: 30-day postsurgical complications	Pearson’s correlation, ANOVA	*Teamwork and cooperation* is associated with higher levels of postsurgical complications	*M*_uncomplicated_ = 3*.*19, *M*_minor complications_ = 3*.*44, *M*_major morbidity_ = 3*.*28, *F*(2,36) = 3*.*85, *p* < 0*.*05, η^2^ = 0*.*18	8*.*5 (16)
Siassakos et al., 2010 [80]	Relationships between individual team members’ knowledge, skills, and attitudes and team performance	no	19 teams (physicians and midwives), 6 maternity units, UK	Video observation of obstetric emergency simulation, self-report questionnaire	Teamwork: SAQ subscale team climate^a^ Patient safety: team performance (magnesium administration)	Kendall’s rank correlation	No correlation between teamwork climate and performance	NS	8 (16)
Siassakos et al., 2011 [72]	Relationships between teamwork skills and behaviors and team performance in emergency situations	yes	47 teams (2 physicians and 4 midwives each), 6 maternity units, UK	Video observation	Teamwork: Team analytical tool^a^ Patient safety: performing key actions	Kendall’s rank correlation	1) Positive correlation between speed of magnesium administration and a) skills b) behavior c) and overall teamwork 2) Negative correlation between time needed to put patient in recovery position and a) skills b) behavior c) but not overall teamwork 3) Negative correlation between time needed to administer oxygen and a) skills b) behavior c) and overall teamwork 4) Negative correlation between time needed to sample blood and a) skills b) behavior c) and overall teamwork	1a) τ = 0*.*54, *p* < 0*.*001 1b) τ = 0*.*41, *p* = 0*.*001 1c) τ = 0*.*51, *p* < 0*.*001 2a) τ = −0*.*29, *p* = 0*.*012 2b) τ = −0*.*25, *p* = 0*.*026 2c) NS 3a) τ = −0*.*39, *p* < 0*.*001 3b) τ = −0*.*28, *p* = 0*.*014 3c) τ = −0*.*41, *p* < 0*.*001 4a) τ = −0*.*35, *p* = 0*.*002 4b) τ = −0*.*35, *p* = 0*.*002 4c) τ = −0*.*35, *p* < 0*.*002	8*.*5 (15)
Siassakos et al., 2011 [71]	Relationships between teamwork and clinical efficiency in emergency situations	yes	114 physicians and nurses, 19 teams, 6 maternity units, UK	Video observation	Teamwork: self-developed observation system Patient safety: performing key action (speed of magnesium administration)	Kendall’s rank correlation	1) Positive correlation between closed-loop communication and clinical efficiency 2) Positive correlation between unambiguous communication and clinical efficiency 3) No correlations between clinical efficiency and a) SBAR communication style b) team coordination c) situational awareness d) leadership style e) supportive language f) task support by senior clinician	1) τ = 0*.*46, *p* = 0*.*022 2) τ = 0*.*53, *p* = 0*.*004 3a) NS 3b) NS 3c) NS 3d) NS 3e) NS 3f) NS	8 (15)
Thomas et al., 2006 [73]	Relationship between teamwork and quality of care	yes	118 teams (physicians, nurses, respiratory therapists), resuscitation room, teaching hospital, USA	Video observation of neonatal resuscitation	Teamwork: Frequency of different teamwork behaviors Patient safety: Neonatal Resuscitation Program (NRP) Guidelines	Spearman’s rank correlation	1) Negative correlation between team communication and a) overall quality of resuscitation, b) non-compliance with all NRP steps, and c) non-compliance during preparation and initial steps 2) Negative correlation between team management and a) noncompliance with all NRP steps, and b) noncompliance during preparation and initial steps but not c) overall quality of resuscitation, 3) Negative correlation between team leadership and a) overall quality of resuscitation, but not with b) noncompliance with all NRP steps, and with c) non-compliance during preparation and initial steps	1a) ρ = −0*.*236, *p* = 0*.*007 1b) ρ = −0*.*214, *p* = 0*.*014 1c) ρ = −0*.*230, *p* = 0*.*008 2a) ρ = −0*.*201, *p* = 0*.*021 2b) ρ = −0*.*252, *p* = 0*.*003 2c) NS 3a) ρ = −0*.*288, *p* < 0*.*001 3b) NS 3c) NS	9*.*5 (15)
Tschan et al., 2006 [74]	Relationships between directive leadership, structuring inquiry and performance regarding different phases	yes	109 clinicians (nurses, residents, senior physicians), 21 teams, ICU, university hospital, Switzerland	Video observation and transcription of emergency simulation	Teamwork: directive leadership and structuring inquiry Patient safety: clinical performance (key actions, hands-on time)	Pearson’s correlation	1) Phase 1 (nurses only): positive correlation between performance and a) directive leadership and b) structuring inquiry 2) Phase 2 (residents and nurses): positive correlation between performance and a) resident directive leadership during first 30 s, no correlation between performance and b) resident directive leadership per second c) resident structuring inquiry per second d) resident structuring inquiry during first 30 s 3) Phase 3 (nurses, residents, senior physicians): positive correlation between performance and a) senior physician structuring inquiry, no correlation between performance and b) resident structuring inquiry c) senior physician d) resident directive leadership	1a) *r* = 0*.*445, *p* < 0*.*05 1b) *r* = 0*.*216, *p* < 0*.*05 2a) *r* = 0*.*522, *p* < 0*.*05 2b) NS 2c) NS 2d) NS 3a) *r* = 0*.*428, *p* < 0*.*01 3b) NS 3c) NS 3d) NS	11*.*5 (15)
Tschan et al., 2009 [75]	Relationships between team communication and perceptual biases of individuals and accuracy of diagnosis	yes	53 physicians, 20 teams, university hospital, Switzerland	Video observation of hand-over simulation	Teamwork: coding of communication and behavior Patient safety: diagnostic performance	ANOVA	1) Groups considering more diagnostic information are not more likely to find the correct diagnosis 2) Groups showing a) more explicit reasoning b) more talking to the room are more likely to find the correct diagnosis	1) NS 2a) *F*(2, 15) = 5*.*750, *p* = 0*.*014 2b) *χ* ^2^ = 8*.*598, *df* = 2, *p =* 0*.*007	11 (15)
Westli et al., 2010 [76]	Relationship between teamwork skills/shared mental models and clinical performance	yes	27 trauma teams, Norway	Video observation of emergency simulations	Teamwork: ANTS^a^ and Anti-Air Teamwork Observation Measure (ATOM) Patient safety: Team global medical management, key actions of trauma management	Pearson’s correlation	1) Negative correlation between supporting behavior and performing key actions 2) Negative correlation between poor coordination and medical management 3) Positive correlation between information exchange and medical management 4) Negative correlation between poor situational awareness and performing key actions 5) Positive correlation between providing information and medical management 6a-u) 21 non-significant correlations between teamwork and patient safety variables	1) *r* = −0*.*37, *p* < 0*.*05 2) *r* = −0*.*36, *p* < 0*.*05 3) *r* = 0*.*34, *p* < 0*.*05 4) *r* = −0*.*40, *p* < 0*.*05 5) *r* = 0*.*51, *p* < 0*.*01 6a-u) NS	10*.*5 (15)
Wiegmann et al., 2007 [77]	Relationship between (teamwork-related) surgical flow disruptions and surgical error	yes	31 cardiac operations, 1 hospital, USA	Live observation of operation	Teamwork: teamwork-related surgical flow disruptions Patient safety: surgical errors	Multiple regression	Teamwork-related surgical flow disruptions positively predict surgical errors	β = 0*.*692, *p* < 0*.*001, adj. *R* ^2^ = 0*.*553, [*f* ^*2*^ = 1*.*24]^bc^ (5 predictors altogether)	11 (15)
Williams et al., 2010 [78]	Relationships between teamwork behaviors and resuscitation errors	yes	12 resuscitation teams, NICU, teaching hospital, USA	Video observation of resuscitation	Teamwork: frequency of different teamwork behaviors Patient safety: Neonatal Resuscitation Program (NRP) Guidelines	Spearman’s rank correlation, generalized linear mixed model (GLM)	1) Negative correlation between vigilance and NRP errors 2) No correlation between workload management and NRP errors 3) NRP errors are associated with a) more assertions before the error b) less teaching after the error 4) No associations between NRP errors and a) information sharing before error b) information sharing after error c) inquiry before error d) inquiry after error e) assertion after error f) teaching before error	1) ρ = −0*.*62, *p* = 0*.*031 2) NS 3a) OR = 1*.*44, *p* = 0*.*008, 95 % CI 1*.*10 – 1*.*89 3b) OR = 0*.*59, *p* = 0*.*028, 95 % CI 0*.*37 – 0*.*94 4a) NS 4b) NS 4c) NS 4d) NS 4e) NS 4f) NS	10 (15)
b) survey studies									
Brewer, 2006 [87]	Relationships between culture, team characteristics/processes and patient safety/hospital financial performance	yes	430 nurses, physicians and other medical care providers, 16 surgical units, 4 acute care hospitals, USA	Cross-sectional self-report questionnaire, record review	Teamwork: Positive team processes: Relational Coordination Scale^a^ Negative team processes scale Patient safety: patient falls (incident reporting system), length of stay (hospital records)	Pearson’s correlation	1) Positive intra-team processes correlate positively with a) length of stay b) but not with patient falls 2) No correlation between negative team processes and a) length of stay b) patient falls	1a) *r* = 0*.*59, *p* < 0*.*05 1b) NS 2a) NS 2b) NS	10 (16)
Chan et al., 2011 [88]	Validity of a team-based tool to assess success of a team-based intervention to reduce central line associated blood stream infections (CLABSI)	no	46 ICUs, 35 hospitals, USA	Secondary analyses of longitudinal RCT, self-report questionnaire, record review	Teamwork: Team check-up tool (TCT) Patient safety: Central line associated bloodstream infections (CLABSI)	Cox regression	No association between teamwork and duration to reach zero CLABSI’s after intervention	NS	10 (19)
Chang & Mark, 2009 [89]	Antecedents (teamwork, nurse & patient factors) of severe and non-severe medication errors	yes	1 671 nurses, 279 units, 146 hospitals, USA	Longitudinal self-report questionnaire, record review	Teamwork: Relational Coordination Scale^a^ Patient safety: medication errors (hospital incident reports)	Generalized estimating equations (GEE)	Relational coordination predicts neither 1) severe nor 2) non-severe medication errors	1) NS 2) NS	9 (16)
Edmondson, 2004 [10]	Relationship between team/organizational characteristics, team leadership and medication errors	yes	159 nurses, physicians and pharmacists, 8 hospitals, USA	Cross-sectional self-report questionnaire, record review	Teamwork: Team/organizational characteristics and team leadership (self-developed questionnaire) Patient safety: medication error (hospital incident reports & self-reported)	Spearman’s rank correlation	Positive correlation between 1) nurse manager coaching 2) nurse manager direction setting and 3) unit relationship quality and a) detected and b) intercepted medication errors but not with c) non-preventable drug complications	1a) ρ = 0*.*74, *p* < 0*.*03 1b) ρ = 0*.*71, *p* < 0*.*03 1c) NS 2a) ρ = 0*.*74, *p* < 0*.*03 2b) ρ = 0*.*83, *p* < 0*.*03 2c) NS 3a) ρ = 0*.*74, *p* < 0*.*03 3b) ρ = 0*.*76, *p* < 0*.*03 3c) NS	11 (16)
Fasolino et al., 2012 [90]	Relationships between nurse characteristics, nurse practice environment, team member effectiveness and medication error	yes	163 nurses, 11 surgical units, 1 hospital, USA	Cross-sectional self-report questionnaire, record review	Teamwork: team member effectiveness survey Patient safety: medication errors (hospital incident reports)	Spearman’s rank correlation	Team member effectiveness is positively correlated with medication error	ρ = 0*.*19, *p* < 0*.*01	12 (16)
Hoffer Gittell et al., 2000 [9]	Relationship between relational coordination and quality of care/length of stay	yes	338 physicians, nurses, and other medical care providers, 9 hospitals, USA	Cross-sectional self-report questionnaire, record review	Teamwork: Relational Coordination Scale^a^ Patient safety: Length of stay	Hierarchical linear regression	Relational coordination is associated with decreased length of stay	*B* = −53*.*77, *p* < 0*.*001, []^b^	13 (16)
Hwang & Ahn, 2015 [83]	Relationships between teamwork and error reporting	yes	576 nurses, 2 acute care hospital, South Korea	Cross-sectional self-report questionnaire	Teamwork: Teamwork perceptions questionnaire (TPQ)^a^ Patient safety: occurrence of and reporting medical errors	Logistic regression	Teamwork dimensions 1) team structure, 2) team leadership, 3) situation monitoring, 4) mutual support, and 5) communication are positively associated with error reporting No information on relationship between teamwork and occurrence of medical errors	1) OR = 0.92, 95 % CI 0.50 –1.692) OR = 1.13, 95 % CI 0.78 –1.623) OR = 0.96, 95 % CI 0.52 – 1.78 4) OR = 1.23, 95 % CI 0.66 – 2.30) 5) OR = 1.82, 95 % CI 1.05 - 3.14	12.5 (16)
Kalisch & Lee, 2010 [60]	Relationship between teamwork and missed nursing care	yes	2216 nurses, 40 acute care units, 4 hospitals, USA	Cross-sectional self-report questionnaire	Nursing Teamwork Survey^a^ MISSCARE Survey^a^	Pearson’s correlation Multiple linear regression	1) Negative correlation between missed nursing care and a) trust b) team orientation c) backup behavior d) shared mental model e) team leadership 2) After controlling for various covariates, overall teamwork scores negatively predict missed nursing care	1a) *r* = −0*.*31, *p* < 0*.*01 1b) *r* = −0*.*28, *p* < 0*.*01 1c) *r* = −0*.*31, *p* < 0*.*01 1d) *r* = −0*.*32, *p* < 0*.*01 1e) *r* = −0*.*29, *p* < 0*.*01 2) *B* = −0*.*254, *p* < 0*.*001, Δ*R* ^*2*^ = 10*.*9, [*f* ^*2*^ = 0*.*124]^b^	12*.*5 (16)
Leroy et al., 2012 [8]	Mediation and moderation relationships between leader behavioral integrity for safety, team psychological safety, team priority of safety, and treatment errors	yes	580 nurses and head nurses, 4 hospitals, Belgium	Longitudinal self-report questionnaire	Teamwork: Team Psychological Safety Scale^a^ Patient Safety: head nurses’ reports of treatment errors	Path analysis	1) Good overall model fit 2) Within path model, team psychological safety at time 1 positively predicts treatment errors at time 2	1) *χ* ^2^ = 6*.*72, *p =* 0*.*03, SRMR = 0*.*07, RMSEA = 0*.*02, CFI = 0*.*98 2) β = 0*.*28, *p* = 0*.*02	14 (16)
Manojlovich et al., 2007 [82]	Relationships between perceived work environments, nurse-physician communication and patient outcomes	yes	462 nurses, 25 ICUs, 8 hospitals, USA	Cross-sectional self-report questionnaire	Teamwork: parts of ICU Nurse-Physician Questionnaire^a^ Patient safety: nurse-reported adverse events (medication errors, ventilator-associated pneumonia, catheter-associated sepsis)	Random intercept multilevel models	Nurse-physician communication negatively predicts 1) ventilator-associated pneumonia 2) catheter-associated sepsis and 3) medication errors	1) *B* = −0*.*045, *p* < 0*.*05, *R* ^*2*^ = 0*.*09, [*f* ^*2*^ = 0*.*1]^b,c^ 2) *B* = −0*.*049, *p* < 0*.*05, *R* ^*2*^ = 0*.*14, [*f* ^*2*^ = 0*.*16]^b,c^ 3) *B* = −0*.*047, *p* < 0*.*01, *R* ^*2*^ = 0*.*11, [*f* ^*2*^ = 0*.*12]^b,c^	
Manojlovich et al., 2009 [91]	Relationship between nurse-physician communication and patient outcomes	yes	462 nurses, 25 ICUs, 8 hospitals, USA	Cross-sectional self-report questionnaire, record review	Teamwork: ICU Nurse-Physician Questionnaire^a^ Patient safety: adverse outcomes ventilator-associated pneumonia, bloodstream infections, and pressure ulcers	Pearson’s correlation	No correlation between nurse-physician communication subscales 1) timeliness 2) accuracy 3) openness and 4) understanding and patient safety indicators a) ventilator-associated pneumonia b) bloodstream infections and c) pressure ulcers	1a-4c) 12 non-significant associations	11 (16)
Ogbolu et al., 2015 [84]	Relationships between nurse work environment and patient safety	no	222 nurses, Nigeria	Cross-sectional self-report questionnaire	Teamwork: nurse-physician relations^a^ Patient safety: Patient safety: one item from AHRQ^a^	Generalized linear mixed modeling	Relationship between nurse-physician relations and patient safety not reported (only relationship between aggregate NWI scale and patient safety)	-	10 (16)
Taylor et al., 2012 [92]	Relationships between safety climate, teamwork and patient adverse events	no	Nurses in 29 units, 1 hospital, USA	Cross-sectional & longitudinal self-report questionnaire, record review	Teamwork: SAQ subscale team climate^a^ Patient safety: patient falls & injuries, deep vein thrombosis and pulmonary embolism records	Multilevel logistic regression	Positive team climate is associated with 1) fewer decubitus ulcers, but not 2) less patient falls & injuries or 3) pulmonary embolisms and deep vein thrombosis one year later	1) OR = 0*.*56, 95 % CI 0*.*30 - 0*.*82, *p* < 0*.*01 2) NS 3) NS	13*.*5 (16)
Vogus et al., 2007 [93]	Moderation of relationship between team safety organizing behaviors and medication errors by trust in manager and existence of care pathways	yes	1033 nurses & 78 nurse managers, 78 units, 10 acute-care hospitals, USA	Cross-sectional self-report questionnaire, record review	Teamwork: Safety Organizing Scale (SOS)^a^ Trust in manager: 2 items Care pathways: 1 item Patient safety: medication errors (number of errors reported to unit’s incident reporting system up to 6 months after survey data collection)	Multilevel Poisson regression	1) Safety organizing negatively predicts medication errors 2) Trust in manager has no impact on reporting of medication errors when level of safety organizing is high. When safety organizing is low and trust in manager is high, more errors are reported 3) Use of care pathways has no impact on reporting of medication errors when safety organizing is low. When safety organizing is high and care pathways are extensively used, fewer errors are reported	1) β = −0*.*29, *p* < 0*.*01, 95 % CI −0*.*57 to −0*.*01 2) β = −0*.*68, *p* < 0*.*001, 95 % CI −1*.*03 to −0*.*32 3) β = −0*.*82, *p <* 0*.*001, 95 % CI −1*.*31 to −0*.*33	13 (16)
Wheelan et al., 2003 [94]	Relationship between teamwork and patient mortality	yes	349 healthcare providers, 17 ICUs, 9 hospitals, USA	Cross-sectional self-report questionnaire, record review	Teamwork: Group Development Questionnaire^a^ Patient safety: Standardized mortality rates	Pearson’s correlation	Level of group development correlates negatively with mortality rates	*r* = −0*.*66, *p* = 0*.*004	12 (16)
Yun et al., 2005 [95]	Moderation of relationship between contingent leadership and team effectiveness by severity of patient trauma and team experience	yes	91 members of trauma resuscitation teams, 1 hospital, USA	Cross-sectional self-report questionnaire, scenario method	Teamwork & patient safety: Team Effectiveness Scale^a^, Team leadership, severity of trauma and team experience manipulated across scenarios	General linear model (GLM)	1) Interaction of leadership/severity of injury: Team effectiveness dimension *quality health care* is high when patient was not severely injured/leadership is empowering or patient was severely injured/leadership was directive 2) Interaction of leadership/team experience: quality health care is highest when leadership is empowering, independent of team experience 3) 3-way-interaction: quality health care is highest when team is experienced and leadership is empowering, independent of patient condition. When team is inexperienced, quality health care is highest when leadership is empowering and patient is not severely injured, or when leadership is directive and patient is severely injured	1) Severely injured patient: *M*_directive leaders_ = 3*.*06, 95 % CI 2*.*83 – 3*.*27, *M*_empowering leaders_ = 2*.*72, 95 % CI 2*.*50 – 2*.*95. Non-severely injured patient: *M*_empowering leaders_ *=* 3*.*91, 95 % CI 3*.*69 – 4*.*13, *M*_directive leaders_ = 2*.*16, 95 % CI 1*.*94 – 2*.*38, *F* = 119*.*48, p < 0*.*01, η^2^ = 0*.*26. 2) Experienced team: *M*_empowering leadership_ = 3*.*65, 95 % CI 3*.*42 - 3*.*82, *M*_directive leadership_ = 2*.*48, 95 % CI 2*.*25 - 2*.*70. Inexperienced team: *M*_empowering leadership_ = 2*.*99, 95%CI 2*.*76 - 3*.*21, *M*_directive leadership_ = 2*.*74, 95 % CI 2*.*51 - 2*.*96, *F* = 23*.*19, *p* < 0*.*01, η^2^ = 0*.*06. 3) Inexperienced team/severely injured patient: *M*_directive leadership_ = 3*.*19, 95 % CI 2*.*89 - 3*.*49, *M*_empowering leadership_ *=* 2*.*13, 95 % CI 1*.*82 - 2*.*44. Inexperienced team/non-severely injured patient: *M*_empowering leadership_ *=* 3*.*85, 95 % CI 3*.*57 - 4*.*12, *M*_directive leadership_ *=* 2*.*28, 95 % CI 2*.*00 - 2*.*56, *F* = 7*.*31, *p* < 0*.*01, η^2^ = 0*.*04.	14*.*5 (16)

We report not only significant but also non-significant relationships between predictor and outcome variables of interest in this review as hypothesized in the reviewed studies; even if not explicitly stated in the original publication

^a^ validated instrument

^b^ effect sizes calculated by authors, calculation not possible if brackets empty

^c^ Cohen’s*ƒ* ^2^ based on R^2^ instead of ΔR^2^

^d^ in brackets: maximal possible score

**Table 4:** Relationships between well-being and patient safety

Study	Topic	Primary topic	Sample & setting	Design & data collection methods	Assessment of variables	Analyses	Findings	Outcomes & effect sizes	Quality score^d^
Arakawa et al., 2011 [98]	Relationships between nurses’ work, health, and lifestyle characteristics and medical errors and incidents	yes	6445 nurses, 99 hospitals, Japan	Cross sectional self-report questionnaire	Well-being: SF-36 scales *mental health* and *vitality* ^a^ Patient safety: Number of incidents and errors during the previous 6 months	Logistic regression	No association between 1) mental health 2) vitality and medical errors and incidents	1) NS 2) NS	9 (16)
Arimura et al., 2010 [99]	Relationships between work characteristics, sleepiness, mental health state and self-reported medical errors	yes	454 nurses, 2 general hospitals, Japan	Cross sectional self- report questionnaire	Well-being: GHQ-28^a^, daytime sleepiness (Epworth sleepiness scale) Patient safety: medical errors during past month	Multiple logistic regression	1) Poorer mental health is associated with higher occurrence of medical errors 2) Daytime sleepiness is not associated with higher occurrence of medical errors	1) OR = 1*.*1, *p* < 0*.*05, 95 % CI 1*.*0 – 1*.*1 2) NS (8 predictors altogether)	105 (16)
Chen et al., 2013 [114]	Relationships between burnout, job satisfaction and medical malpractice	yes	809 physicians, Taiwan	Cross-sectional self-report questionnaire	Well-being: MBI^a^ Patient safety: experiences of medical malpractice	Univariate logistic regression	1) Emotional exhaustion is associated with higher risk of medical malpractice, whereas 2) depersonalization and 3) personal accomplishment are associated with lower risk of medical malpractice	1) OR = 1.50, 95 % CI 0.68 –1.95 2) OR = 0.74, 95 % CI 0.40 –1.36 3) OR = 0.76, 95 % CI 0.07 –1.05	6 (16)
Cimiotti et al., 2012 [104]	Relationships between nurse staffing, burnout, and hospital infections	yes	7076 nurses, 161 hospitals, USA	Cross-sectional self-report questionnaire, hospital records	Well-being: MBI^a^ Patient safety: catheter-associated urinary tract & surgical site infections	Linear regression	Burnout is positively associated 1) catheter-associated urinary tract and 2) surgical site infections	1) β = 0.82, *p* < .05 2) β = 1.56, *p* < .01	10.5 (16)
Fahrenkopf et al., 2008 [106]	Relationships between depression, burnout, and medication errors	yes	123 residents, 3 pediatric hospitals, USA	Cross-sectional self-report questionnaire, record review	Well-being: MBI^a^ Patient safety: medical errors (self-report & chart reviews)	Cluster adj. Poisson analysis, Fisher’s exact test	1) Burnt out residents perceive their number of errors to be higher than residents who are not burnt out 2) Burnt out residents are more likely to attribute errors to sleep deprivation 3) No significant differences in error rates detected in chart reviews between both groups	1) *M*_high burnout_ = 2*.*3, *M*_low burnout_ = 1*.*0, *p* = 0*.*002 2) 29 % vs. 10 %, *p* = 0*.*05 3) NS []^b^	8 (16)
Garrouste-Orgeas et al., 2015 [116]	Relationships between medical errors, burnout, depression, and safety culture	yes	1534 nurses, physicians, & other healthcare staff, 31 ICUs, France	Cross-sectional self-report questionnaire, hospital records and observations	Well-being: MBI^a^ Patient safety: Medical error	Negative binomial regression	Burnout is not associated with medical error	NS	10.5 (15)
Halbesleben et al., 2008 [22]	Relationships between nurse burnout and patient safety perceptions/reporting behavior	yes	148 nurses, 1 hospital, USA	Cross sectional self- report questionnaire	Well-being: Emotional Exhaustion and Depersonalization^a^ Patient safety: AHRQ Patient Safety Culture Survey^a^ & frequency of incident reports	Multiple linear regression	1) Emotional exhaustion and depersonalization predict patient safety dimensions a) safety grade b) safety perception c) near-miss reporting frequency 2a) Emotional exhaustion and b) depersonalization do not predict patient safety dimension event reports	1a) β_exhaustion_ = −0*.*40, *p* < 0*.*01, β_depersonlization_ = −0*.*16, *p* < 0*.*05, *R* ^*2*^ = 0*.*22, [*f* ^*2*^ = 0*.*28]^b,c^ 1b) β_exhaustion_ = −0*.*84, *p <* 0*.*001, β_depersonlization_ = −0*.*26, *p* < 0*.*05, *R* ^*2*^ = 0*.*36, [*f* ^*2*^ = 0*.*56]^b, c^ 1c) β_exhaustion_ = −0*.*14, *p <* 0*.*05, β_depersonlization_ = −0*.*36, *p* < 0*.*01, *R* ^*2*^ = 0*.*18, [*f* ^*2*^ = 0*.*22]^b, c^ 2a) NS 2b) NS	13.5 (16)
Halbesleben & Rathert, 2008 [107]	Relationship between physician burnout and patient satisfaction and patient recovery time after hospital discharge	yes	178 patient and physician dyads, 1 hospital, USA	Cross-sectional self- report questionnaire	Well-being: MBI^a^, patients’ perception of physician depersonlization Patient safety: recovery time: 1-item patient self-report	Path analysis, Pearson’s correlation	1) Good overall model fit 2) Positive correlation between patient recovery time and a) depersonalization b) but not emotional exhaustion c) or personal accomplishment 3) Positive correlation between patients’ perception of physician depersonalization and recovery time 4) No correlation between physician emotional exhaustion and recovery time	1) GFI = 0*.*99, CFI = 1*.*00, NNFI = 1*.*02, AIC = −2*.*98, BIC = −8*.*45, RMSEA = 0*.*00 2a) *r* = 0*.*44, *p* < 0*.*05 2b) NS 2c) NS 3) *r* = 0*.*32, *p* < 0*.*05 4) NS	12 (16)
Hayashino et al., 2012 [108]	Hope moderates relationship between distress and medical errors	yes	836 physicians, Japan	Longitudinal self-report questionnaire	Well-being: MBI^a^ (time 1) Medical errors: self-report (time 2)	Poisson regression	High scores in 1) emotional exhaustion 2) depersonalization and low scores in 3) personal accomplishment at time 1 are associated with medical errors at time 2	1) IRR = 2*.*34, *p* < 0*.*0001 2) IRR = 2*.*72, *p* < 0*.*0001 3) IRR = 0*.*62, *p* = 0*.*001	9*.*5 (16)
Hunziker et al., 2012 [109]	Influence of self-reported, biochemical and physiological stress on cardio-pulmonary resuscitation (CPR) performance	yes	28 residents, teaching hospital, Switzerland	Self-report questionnaire, video observation of simulated resuscitation	Well-being: Stress/overload index (self-report; blood cortisol, heart rate) Patient safety: performance (time until CPR is started and hands-on time)	Multiple linear regression	1) Stress/overload is positively associated with a) time to start CPR b) but not hands-on-time during resuscitation 2) Heart rate is positively associated with a) hands-on-time b) and negatively with time to start CPR during resuscitation 3a) Cortisol level and b) heart rate variability do not predict c) hands-on-time and d) time to start CPR 4) The difference of a) stress/overload b) cortisol level c) heart rate variability before to during resuscitation do not predict d) hands-on-time or e) time to start CPR 5) The difference of heart rate before to during resuscitation predicts a) hands-on-time and b) time to start CPR	1a) β/*B* = 12*.*01, 95 % CI 0*.*65 – 23*.*36, *p* = 0*.*04 1b) NS 2a) β/*B* = 2*.*22, 95 % CI 0*.*53 – 3*.*92, *p* = 0*.*015 2b) β/*B* = −0*.*78, 95 % CI 1*.*44 to −0*.*11, *p* = 0*.*027 3 ac) NS 3ad) NS 3bc) NS 3bd) NS 4ad) NS 4ae) NS 4bd) NS 4be) NS 4 cd) NS 4ce) NS 5a) β/*B* = 2*.*73, 95 % CI 0*.*48 – 4*.*99, *p* = 0*.*022 5b) β/*B* = −1*.*12, 95 % CI −1*.*91 to −0*.*33, *p* = 0*.*01 (no information regarding standardization of coefficients)	12.5 (15)
Jones et al., 2012 [100]	Effect of incident seriousness and work-based support on negative positive affect	yes	171 nurses, 4 hospitals, UK	Cross-sectional & longitudinal between & within-person design, diary study	Well-being: Positive & Negative Affect Scale (PANAS) and mood diary entries^a^ Patient safety: nurse-reported incidents	Random-effects multilevel model	1) Interaction of incident occurrence and seriousness leads to elevated negative affect during remainder of shift 2a) Incident occurrence 2b) but not incident seriousness lead to reduced positive affect during remainder of shift	1) β = 0*.*07, *z* = 3*.*5, *p* < 0*.*005 2a) β = −2*.*39, *z* = 1*.*99, *p* < 0*.*05 2b) NS	13 (16)
Kirwan et al., 2013 [105]	Relationships between working environment, burnout and patient safety	no	1397 nurses, 108 wards, 30 hospitals, Ireland	Cross-sectional self-report questionnaire	Well-being: MBI^a^ Patient safety: one item from AHRQ^a^, adverse events	Multilevel regression	Emotional exhaustion on ward level does not predict 1) nurse-rated patient safety or 2) reporting of adverse events	1) NS 2) NS	12.5 (16)
Klein et al., 2010 [110]	Relationship between burnout and self-reported quality of care	yes	1311 surgeons, 489 hospitals, Germany	Cross sectional self- report questionnaire	Well-being: Copenhagen Burnout Inventory (CBI^a^) Patient safety: Quality of care: frequency of diagnostic and therapeutic errors (Chirurgisches Qualitätssiegel survey CQS)	Multivariate logistic regression	1) Burnout is associated with 1a) lower quality of diagnosis/therapy 1b) more diagnostic errors 1c) more therapeutic errors among males 2) Unclear association of burnout with 2a) lower quality of diagnosis/therapy 2b) more diagnostic errors 2c) more therapeutic errors among females	1a) OR = 1*.*71, 95 % CI 1*.*10 – 2*.*64 1b) OR = 1*.*94, 95 % CI 1*.*35 – 2*.*79 1c) OR = 2*.*56, 95 % CI 1*.*66 – 3*.*96 2a-c) contradictory information regarding significance in text and table	10.5 (16)
Maiden et al., 2011 [101]	Relationship between moral distress, compassion fatigue, and causes of medication errors	yes	205 nurses, ICU, USA	Cross sectional self-report questionnaire, focus group	Well-being: Moral distress scale^a^ Compassion fatigue: Professional Quality of Life Scale^a^ Patient safety: Medication Administration Error Survey^a^	Pearson’s correlation	1) Positive correlation between moral distress and a) transcription related medication errors and b) physician communication related medication errors c) but not with medication packaging d) pharmacy processes 2) Compassion fatigue is positively correlated with a) transcription related medication errors but not with medication error due to b) physician communication c) medication packaging d) pharmacy processes	1a) *r* = 0*.*20, *p* = 0*.*05 1b) *r* = 0*.*24, *p* = 0*.*01 1c) NS 1d) NS 2a) *r* = 0*.*15, *p* = 0*.*05 2b) NS 2c) NS 2d) NS	9 (16)
Merlani et al., 2011 [13]	Relationships between hospital, patient, and clinician characteristics and burnout/stress	yes	3052 physicians, nurses, and nurse-assistants, 74 ICUs, Switzerland	Cross-sectional self-report questionnaire, record review	Well-being: MBI^a^, 1 stress item Patient safety: mortality rates and length of stay (unit records)	Multivariate logistic regression	1) Mortality is associated with higher level of burnout 2) Length of stay is not associated with burnout	1) OR = 1*.*060, *p* = 0*.*04, 95 % CI 1*.*003 – 1*.*120 2) NS	12.5 (16)
Prins et al., 2009 [97]	Relationships between self-reported errors, burnout, and engagement	yes	2115 residents, The Netherlands	Cross-sectional self- report questionnaire	Well-being: Utrecht Burnout Scale (UBOS)^a^, Utrecht Work Engagement Scale (UWES)^a^ Patient safety: medical errors	Pearson’s correlation	1) Errors due to wrong actions/inexperience a) are positively correlated with emotional exhaustion b) depersonalization c) and negatively correlated with personal accomplishment 2) Errors due to wrong actions/inexperience are not correlated with d) vigor e) dedication f) absorption 3) Errors due to lack of time a) are positively correlated with emotional exhaustion b) depersonalization c) and negatively correlated with personal accomplishment 4) Errors due to lack of time are negatively correlated with a) vigor b) dedication c) absorption	1a) *r* = 0*.*20, *p* < 0*.*001 1b) *r* = 0*.*29, *p* < 0*.*001 1c) *r* = −0*.*05, *p* < 0*.*001 2a) NS 2b) NS 2c) NS 3a) *r* = 0*.*43, *p* < 0*.*001 3b) *r* = 0*.*42, *p* < 0*.*001 3c) *r* = −0*.*08, *p* < 0*.*001 4a) *r* = −0*.*23, *p* < 0*.*001 4b) *r* = −0*.*24, *p* < 0*.*001 4c) *r* = −0*.*11, *p* < 0*.*001	10.5 (16)
Ramanujam et al., 2008 [102]	Relationship between nurses’ work characteristics, burnout, and patient safety	yes	430 nurses, 2 hospitals, USA	Cross sectional self- report questionnaire	Well-being: Not described, although it can be deducted from the paper that the MBI^a^ was used Patient safety: nurses’ safety perception	Path analysis	1) Unsatisfactory initial model fit statistics, final model statistics not reported 2) Positive association between depersonalization and perceived patient safety 3) No association between emotional exhaustion and perceived patient safety	1) *χ* ^2^ = 1100*.*60, *df* = 455, *χ* ^2^ */df* = 2*.*419, CFI = 0*.*876, RMSEA = 0*.*058 2) β = 0*.*189, *p* < 0*.*001 3) NS	8*.*5 (16)
Shanafelt et al., 2002 [112]	Prevalence of burnout in medical residents and the relationship to self-reported patient care practices	yes	115 internal medicine residents, USA	Cross sectional self- report questionnaire	Well-being: MBI^a^ Patient safety: self-developed patient care practices measure	Stepwise logistic regression	1) Overall burnout score is associated with higher levels of a) monthly b) weekly suboptimal patient care practices 2) Depersonalization is associated with higher levels of a) monthly b) weekly suboptimal patient care practices 3) No associations between a) emotional exhaustion b) personal accomplishment and c) monthly d) weekly suboptimal patient care practices	1a) OR = 8*.*3, *p* < 0*.*001, 95 % CI 2*.*6 – 26*.*5 1b) OR = 4*.*0, *p* = 0*.*036, 95 % CI 1*.*1 – 14*.*2 2a) OR = 5*.*8, *p* < 0*.*001, 95 % CI 2*.*2 – 15*.*4 2b) OR = 2*.*8, *p* = 0*.*041, 95 % CI 1*.*1 – 7*.*7 3 ac) NS 3ad) NS 3bc) NS 3bd) NS	10.5 (16)
Shanafelt et al., 2010 [111]	Relationship between burnout, quality of life, depression and perceived major medical errors	yes	7905 surgeons, USA	Cross sectional self- report questionnaire	Well-being: MBI^a^ Patient safety: medical errors	Logistic regression	1a) Emotional exhaustion and b) depersonalization are associated with higher odds of reporting an error 2) Personal accomplishment is associated with lower odds of reporting an error	1a) OR = 1*.*048, *p* < 0*.*0001, 95 % CI 1*.*042 – 1*.*055 1b) OR = 1*.*109, *p* < 0*.*0001, 95 % CI 1*.*096 – 1*.*122 2) OR = 0*.*965, *p* < 0*.*0001, 95 % CI 0*.*955 – 0*.*975	9 (16)
Squires et al., 2010 [103]	Relationships between nurse leadership, work environment, safety climate, and nurse and patient outcomes	no	600 acute care nurses, USA	Cross sectional self- report questionnaire	Well-being: Emotional Exhaustion^a^ Patient safety: medication errors and ulcers	Path analysis	1) Very good final model fit 2) No association between pressure ulcers and emotional exhaustion 3) Positive association between medication errors and emotional exhaustion	1) *χ* ^2^ = 217*.*6, *p* < 0*.*001, SRMR = 0*.*054, CFI = 0*.*947, RMSEA = 0*.*047, PCLOSE = 0*.*67 2) NS 3) β = 0*.*14, *p* < 0*.*05	12 (16)
Teng et al., 2010 [14]	Interactions between time pressure and burnout on patient safety	yes	458 nurses, 90 units, 2 medical centers, Taiwan	Cross sectional self- report questionnaire	Well-being: MBI^a^ Patient safety: frequency of adverse events scale	Multiple linear regression	1) Burnout negatively predicts patient safety 2) The interaction of burnout and time pressure negatively predict adverse events	1) β = −0*.*25, *p* = 0*.*00 2) β = −0*.*08, *p* = 0*.*03 *R* ^*2*^ = 0*.*06 [*f* ^*2*^ = 0*.*06]^b, c^ (7 predictors altogether)	13 (16)
Welp et al., 2015 [117]	Relationships between burnout, demographic and unit characteristics, and patient safety	yes	1425 nurses and physicians, 54 intensive care units, Switzerland	Cross-sectional self-report questionnaire, hospital records	Well-being: MBI^a^ Patient safety: standardized mortality ratios, length of stay, clinician-rated patient safety	Hierarchical (multilevel) linear regression	1a) Emotional exhaustion and 1b) depersonalization are negatively associated with clinician-rated patient safety; c) personal accomplishment is positively associated with clinician-rated patient safety 2a) Emotional exhaustion, but not 2b) depersonalization or 2c) personal accomplishment is positively associated with standardized mortality ratios 3a) Emotional exhaustion, 3b) depersonalization, and 3c) personal accomplishment are not associated with length of stay	1a) B = −0.13, *p* < .001 1b) B = −0.07, *p* < .05 1c) B = 0.16, *p* < .01 2a) β = 0.39, *p* < .05 2b) NS 2c) NS 3a) NS 2b) NS 2c) NS	15 (16)
West et al., 2006 [113]	Relationships between distress, quality of life and medical errors	yes	184 internal medicine residents, teaching hospital, USA	Longitudinal cohort study, self-report questionnaire	Well-being: MBI^a^, fatigue and sleepiness: 2 items Patient safety: medical errors	Generalized estimation equations (GEE)	1) Higher levels of a) emotional exhaustion b) depersonalization are associated with major medical errors in the c) previous d) following 3 months 2) Lower levels of personal accomplishment are associated with higher levels of major medical error in the a) previous b) following 3 months	1 ac) PE = 4*.*58, *p* = 0*.*002 1bc) PE = 2*.*45, *p* = 0*.*002 1ad) OR = 1*.*07, *p* = < 0*.*001, 95 % CI 1*.*03 – 1*.*12 1bd) OR = 1*.*10, *p* = 0*.*001, 95 % CI 1*.*04 – 1*.*16 2a) PE = −2*.*59, *p* = 0*.*002 2b) OR = 0*.*93, *p* = 0*.*02, 95 % CI 0*.*88 – 0*.*99	12 (16)
West et al., 2009 [23]	Relationships between fatigue, distress, and medical errors	yes	380 internal medicine residents, teaching hospital, USA	Longitudinal cohort study, self-report questionnaire	Well-being: MBI^a^, fatigue and sleepiness: 2 items Patient safety: medical errors	Generalized estimation equations (GEE)	Higher levels of 1) sleepiness 2) fatigue 3) emotional exhaustion 4) depersonalization and 5) lower levels of personal accomplishment are associated with subsequent medical errors	1) OR = 1*.*10, *p* = 0*.*002, 95 % CI 1*.*03 – 1*.*16 2) OR = 1*.*14, *p* < 0*.*001, 95 % CI 1*.*08 – 1*.*21 3) OR = 1*.*06, *p* < 0*.*001, 95 % CI 1*.*04 – 1*.*08 4) OR = 1*.*09, *p* < 0*.*001, 95 % CI 1*.*05 – 1*.*12 5) OR = 0*.*94, *p* < 0*.*001, 95 % CI 0*.*92 – 0*.*97	13 (16)
Wetzel et al., 2010 [115]	Relationships between stress and surgical performance	yes	30 surgeons, 1 hospital, UK	Cross-sectional self-report questionnaire, observation of simulated operations	Well-being: State-Trait Anxiety Inventory (STAI)^a^, heart rate, cortisol, oberver rating Patient safety: OTAS^a^, End Product Assessment Rating Scale (EPA)	Linear regression	Non-crisis simulation: No relationship between 1) STAI 2) heart rate 3) cortisol 4) observer stress rating and a) OTAS b) EPA Crisis simulation: 5) no results reported on relationships between the above variables 6) Interaction between low experience and “stress” (not clear how variable was calculated) predicts lower a) EPA and b) OTAS	1a) NS 1b) NS 2a) NS 2b) NS 3a) NS 3b) NS 4a) NS 4b) NS 5) N/A 6a) β = .54, *p* < .01 6b) β = .65, *p* < .001	10 (15)

We report not only significant but also non-significant relationships between predictor and outcome variables of interest in this review as hypothesized in the reviewed studies; even if not explicitly stated in the original publication

^a^validated instrument

^b^effect sizes calculated by authors, calculation not possible if brackets empty

^c^Cohen’s*ƒ* ^2^ based on R^2^ instead of ΔR^2^

^d^in brackets: maximal possible score

**Table 5:** Relationships between teamwork, well-being and patient safety

Study	Topic	Primary topic	Sample & Setting	Design & data collection methods	Assessment of variables	Analyses	Findings	Outcomes & effect sizes	Quality score^b^
Davenport et al., 2007 [118]	Relationships between team and safety climate, working conditions, emotional exhaustion and patient morbidity/mortality	yes	6083 surgical team members, 52 hospitals, USA	Cross-sectional self-report questionnaire, record review	Teamwork: SAQ subscale team climate^a^, levels of communication and collaboration Well-being: Emotional Exhaustion^a^ Patient safety: risk adjusted 30-day morbidity/mortality	Spearman’s rank correlation	1) Negative association between patient morbidity and a) clinician’s communication with attending doctors b) but not with clinician’s communication with residents c) nurses d) other health care providers 2) No associations between team climate and a) mortality b) morbidity 3) No associations between emotional exhaustion and a) mortality b) morbidity	1a) ρ = −0.38, *p* < 0.01 1b) NS 1c) NS 1d) NS 2a) NS 2b) NS 3a) NS 3b) NS	11*.*5 (16)
Laschinger & Leiter, 2006 [119]	Mediation of relationship between nursing work environment and patient safety outcomes by burnout	yes	8597 nurses, acute care hospitals, Canada	Cross-sectional self- report questionnaire	Teamwork: Nurse-Physician-Relations Scale^a^ Well-being: MBI^a^ Patient safety: adverse events scale	Path analysis	1) Good overall model fit 2) Nurse-physician-relations and a) emotional exhaustion b) depersonalization c) adverse events are negatively correlated d) personal accomplishment are positively correlated 3) Adverse events and a) emotional exhaustion b) depersonalization are positively correlated c) personal accomplishment are negatively correlated (only results from correlation matrix are reported)	1) *χ* ^2^ = 16 438.19, *df* = 1.344, CFI = 0.908, IFI = 0.908, RMSEA = 0.037 2a) *r* = −0.22, *p* = <0*.*01 2b) *r* = −0*.*16, *p* = <0*.*01 2c) *r* = −0*.*14, *p* = <0*.*01 2d) *r* = 0*.*13, *p* = <0*.*01 3a) *r* = 0*.*30, *p* = <0*.*01 3b) *r* = 0*.*34, *p* = <0*.*01 3c) *r* = −0*.*22, *p* = <0*.*01	10*.*5 (16)
Rathert et al., 2009 [120]	Mediation of relationships between work environment and work engagement, commitment and patient safety by psychological safety	no	306 nurses and other clinical care providers, acute care hospital, USA	Cross-sectional self-report questionnaire	Teamwork: Psychological Safety Scale^a^ Well-being: Work engagement scale Patient safety: scale adapted from AHRQ Patient Safety Culture Survey	Path analysis	1) Good overall model fit 2) Psychological safety does not mediate relationship between work environment and a) patient safety b) work engagement 3) Positive correlation between patient safety and a) work engagement b) psychological safety 4) No correlation between psychological safety and work engagement	1) RMSEA = 0*.*06, NNFI = 0*.*92, CFI = 0*.*93 2a) NS 2b) NS 3a) *r* = 0*.*14, *p* > 0*.*013 3b) *r* = 0*.*39, *p* < 0*.*01 4) NS	10*.*5 (16)
Van Bogaert et al., 2014 [122]	Relationships between nurse practice environment, work characteristics, burnout and job and patient outcomes	no	1108 nurses, 96 units, 7 hospitals, Belgium	Cross-sectional self-report questionnaire	Teamwork: nurse-physician relations^a^ Well-being: MBI^a^ Patient safety: patient falls, hospital-acquired infections, medication errors	Multilevel regression	1) Good nurse-physician relations on the unit level are associated with fewer a) patient falls b) hospital-acquired infections and c) medication errors 2) Emotional exhaustion on the unit level is associated with more a) patient falls b) hospital-acquired infections and c) medication errors 3) Depersonalization on the unit level is associated with more a) patient falls b) hospital-acquired infections and c) medication errors 4) High personal accomplishment on the unit level is associated with fewer a) patient falls b) hospital-acquired infections and c) medication errors	1a) Adj. OR = 0.70, 95 % CI 0.48 – 1.03 1b)) Adj. OR = 0.56, 95 % CI 0.41 – 0.78 1c) Adj. OR = 0.58, 95 % CI 0.41 – 0.82 2a) Adj. OR = 1.25, 95 % CI 1.06 – 1.48 2b) Adj. OR = 1.33, 95 % CI 1.15 – 1.53 2c) Adj. OR = 1.39, 95 % CI 1.20 – 1.61 3a) Adj. OR = 1.40, 95 % CI 1.15 - 1.70 3b) Adj. OR = 1.57, 95 % CI 1.31 - 1.87 3c) Adj. OR = 1.67, 95 % CI 1.40 - 2.00 4a) Adj. OR = 0.81, 95 % CI 0.64 - 1.02 4b) Adj. OR = 0.78, 95 % CI 0.64- 0.95 4c) Adj. OR = 0.88, 95 % CI 0.71 - 1.08	12.5 (16)
Wilkins et al., 2008 [121]	Relationships between nurses’ work environment, health status and medication errors	no	4379 nurses, Canada	Cross-sectional self-report, phone interviews	Teamwork: Nurse-Physician-Relations Scale^a^ Well-being: mental health (1 item) Patient safety: medication error (1 item)	Logistic regression	1) Lower levels of nurse-physician relations are associated with more medication errors 2) Mental health status is not associated with medication errors	1) OR = 1*.*6, 95 % CI 1*.*1 – 2*.*3, *p* < 0*.*05 2) NS	11 (16)

We report not only significant but also non-significant relationships between predictor and outcome variables of interest in this review as hypothesized in the reviewed studies; even if not explicitly stated in the original publication

^a^ validated instrument

^b^ in brackets: maximal possible score

### Relationships between teamwork and clinician occupational well-being

#### Design & sample

**Fig. 2:**
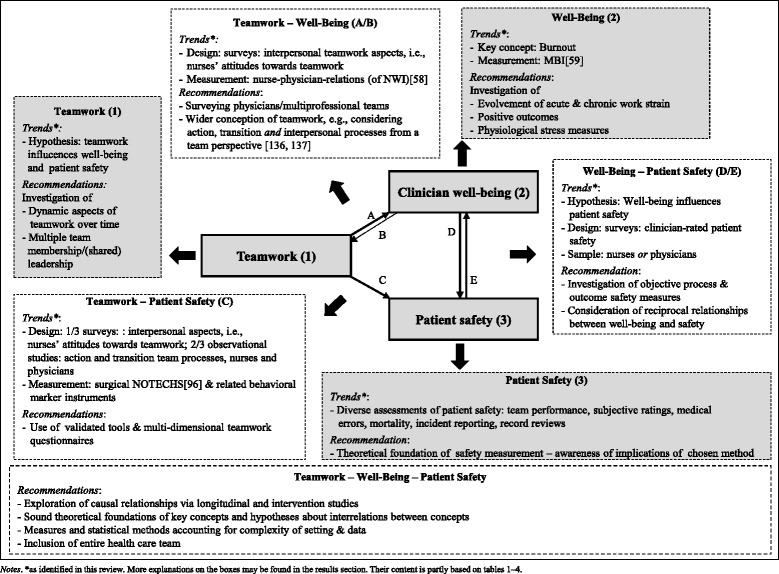
Integrative framework of teamwork, clinician occuptional well-being and patient safety in hospital settings *Notes*. *as identified in this review. More explanations on the boxes may be found in the results section. Their content is partly based on Tables 1, 2, 3 and 4

Out of 25 studies examining relationships between teamwork and clinician occupational well-being, 24 (96 %) used cross-sectional self-report designs, with one study adding a pre-post-shift diary design (Table 2 and box A/B in Fig. 2) [18]. One study employed a longitudinal self-report design [37]. Of these 25 studies, 19 (76 %) surveyed only nurses [18–20, 37–52], one (4 %) physicians [21], one (4 %) midwives [53], and four (16 %) included a mixed sample [54–57].

#### Measures

Studies operationalized teamwork most often with the nurse-physician-relations subscale of the Nursing Work Index (NWI; 12 studies/48 %) [18, 19, 41, 42, 44, 46, 47, 50, 52, 54, 55, 58]; and clinician occupational well-being with the Maslach Burnout Inventory or short versions thereof (MBI; 11 studies/44 %; see box A/B and box 2 in Fig. 2) [19, 38, 41–47, 55, 59].

#### Findings

Studies examining relationships between teamwork and well-being focused on interpersonal teamwork aspects (box A/B in Fig. 2). Most authors assumed that teamwork, a variable inherent to the working context, influences individuals’ *general* occupational well-being, rather than well-being influencing teamwork. Two studies (8 %) focused on *acute* strain [18, 54] one of which showed that it was negatively associated with team behaviors such as closed-loop communication or backup behavior [54, 60]. The only longitudinal study reported an effect of teamwork at time 1 on well-being at time 2. However, since this study did not conduct comprehensive analyses (i.e., testing for reverse causal relationships), we could not draw definite conclusions regarding causal relationships between teamwork and clinician occupational well-being [37].

Out of 25 studies examining relationships between teamwork and clinician occupational well-being, 19 (76 %) focused on interpersonal team processes in rather stable nursing teams, such as nurses’ perceptions of interprofessional teamwork or team cohesion [18–21, 37, 39, 41–47, 49–53, 57]. Four studies (20 %) did not address specific aspects of teamwork, but measured it on a general level [38, 40, 48, 54, 55]. One study (4 %) included a short questionnaire on all three team processes (i.e., action, transition, and interpersonal) [56].

Some studies examined the larger clinical work context without formulating assumptions about the specific relationships between teamwork and clinician occupational well-being, the respective findings thus being a by-product of the larger study context rather than a focus of investigation (see column ‘primary topic’ in Table 2). Across the 25 studies investigating associations between teamwork and clinician occupational well-being, 48 out of 62 (77 %) relationships reported were significant and matched author’s assumptions. Of these significant relationships, 15 (31 %) showed a positive association between both positive indicators of teamwork and well-being (e.g., work engagement), whereas 33 (69 %) showed a negative association between positive indicators of teamwork and negative indicators of well-being (e.g., burnout). Out of the 14 non-significant associations, six (43 %) were in accordance with hypotheses (i.e., teamwork on the hospital level is not related to individual burnout) [50]. Thus, overall findings indicate that clinicians perceiving higher quality of teamwork also reported higher occupational well-being or less strain. Effect sizes ranged from small (β = −12.85; *f*^*2*^ = 0.13) to medium (*r* = −0.47, Table 2).

### Relationships between teamwork and patient safety

#### Design & sample

Studies examining relationships between teamwork and patient safety were very diverse regarding study design, construct operationalization, setting, data collection methods and strength of statistical relationships (see Table 3). Of 43 studies, 25 (58 %) employed video- or live-observation of nurses and physicians in real or simulated acute clinical situations (Table 3a) [5, 6, 11, 12, 61–81]. Five studies (12 %) utilized cross-sectional designs with self-report questionnaires (Table 3b and box C in Fig. 2) [8, 60, 82–84]. Another 13 studies (30 %) employed mixed-method designs (e.g., record reviews or observations plus questionnaires) [9, 10, 85–95]. These studies included one intervention (2 %) [88] and three studies (7 %) with longitudinal aspects [8, 88, 89]. Of the studies using questionnaires seven (16 %) surveyed either nurses [60, 82, 89–93] and seven (16 %) surveyed a mixed sample [9, 10, 83, 84, 87, 94, 95]. Observational studies, in contrast, analyzed teams usually consisting of nurses, physicians (and other healthcare professionals) with the exception of four studies (9 %) [11, 70, 75, 81].

#### Measures

Observational studies most frequently used the Surgical NOTECHS tool (a tool to observe non-technical skills or team behaviors in acute care settings; see box C in Fig. 2) [96] and its adaptations to various clinical settings to assess teamwork (21 %) [6, 62–64, 69, 70, 76, 85, 86]. Studies assessed patient safety using subjective ratings (6 studies/16 %) [8, 60, 82–84, 95], indicators based on hospital records (13 studies/30 %) [9, 74, 75, 85–94] and observational data (22 studies/52 %) [5, 6, 11, 12, 61–70, 73, 77–81, 85, 86]. These observational studies often used execution of key treatment actions (i.e., steps in the care process that are considered indispensable for successful treatment in potentially life threatening situations, such as the administration of magnesium sulfate for eclampsia) as a proxy measure for patient safety (10 studies/23 %) [11, 65, 68, 70–73, 79–81]. Only one study utilized both objective and subjective patient safety indicators [10].

#### Findings

Overall, findings were rather inconsistent for the relationship between teamwork and patient safety. All authors assumed that teamwork positively influenced patient safety. A longitudinal study confirmed this assumption (box 1 in Fig. 2) [8]. In the 43 studies investigating teamwork and patient safety, authors reported 239 relationships, 105 (44 %) of which were significant. The majority of survey and observational studies (23/53 %) reported positive associations between teamwork and patient safety [5, 6, 9, 12, 60, 61, 63–65, 67, 69–77, 79, 82, 92, 94, 95]. In line with this, the valence of 198 (83 %) of the 239 significant associations matched authors’ anticipations (i.e., a positive correlation between both positive indicators of teamwork and patient safety, such as coordination and clinical performance, or negative correlation between a positive indicator of teamwork and a negative indicator of patient safety, such as errors). However, the valence of 41 associations (17 %) was not in line with assumptions (i.e., a negative correlation between positive indicators of both teamwork and patient safety or a positive correlation between positive indicators of teamwork and negative indicators of patient safety). Thus, eight studies (19 %) contained findings suggesting that better teamwork was seemingly associated with lower patient safety [8, 10, 11, 62, 76, 85–87, 90]. Some of these findings may have been coincidental, but the majority may be explained by study design. In survey studies on medical errors, instead of the number of errors, authors measure participants’ propensity to report errors, which in turn may be fostered by positive interpersonal team relationships. In a similar vein, positive associations between teamwork and unfavorable patient outcomes like complications or operative duration in observational studies may simply reflect the necessity for increased coordinative behaviors in complicated cases (box 3 in Fig. 2). Moreover, studies investigating links between teamwork and objective or observational patient safety indicators were frequently unable to identify significant relationships (Table 3a, b). For example, two studies (5 %) used a sample of clinicians surveyed with a teamwork questionnaire to examine associations with objective and subjective patient safety indicators [82, 91]. While no association between teamwork and preventable adverse events extracted from hospital records was found [91], the effect was significant when using the frequency of these events reported by lead nurses [82].

Studies using observational tools to investigate teamwork in relation to patient safety focused on action and transition processes with nine (33 %) of altogether 27 studies examining just action processes [5, 11, 12, 61, 68, 74, 76, 77, 79], and six (22 % of observational studies) measuring both [62, 66, 67, 71–73]. Eight observational studies (30 %) measured action, transition, and interpersonal processes without clear distinction between these dimensions [6, 63, 64, 69, 70, 78, 85, 86]. Two observational studies (7 %) focused on interpersonal processes only [65, 80]. One study (4 %) examined transition processes [75]; and one study (4 %) did not provide further details on the teamwork measure [81].

Studies using questionnaires to examine teamwork in relation to patient safety were rather diverse with regard to teamwork processes. The largest part examined teamwork in general, with no clear distinction between action, transition, and interpersonal processes (8 studies/44 % of survey studies) [9, 60, 83, 87–90, 95],. followed by a focus on interpersonal processes (e.g., team climate or nurse-physician relations; 5 studies, 27 %) [8, 82, 84, 91, 92]. Two studies examined interpersonal and transition processes (13 %) [10, 94], and one study examined action and transition processes (6 %) (again, with no clear distinction between these dimensions) [93].

Effect sizes ranged from small (*r* = −0.08) to large (*r* = −0.66, Tables 3a, b).

### Relationships between clinician occupational well-being and patient safety

#### Design & sample

The majority of the 25 studies examining relationships between clinician occupational well-being and patient safety (Table 4) targeted either nurses (10 studies/40 %) [14, 22, 98–105] *or* physicians (12 studies/48 %; box D/E in Fig. 2) [23, 97, 106–115], with only three studies (12 %) using a mixed sample [13, 116, 117]. Twenty studies (80 %) employed a cross-sectional design [13, 14, 22, 97–99, 101–107, 109–112, 114, 116, 117] and four (16 % used a design with longitudinal aspects [23, 100, 108, 113]. One study (4 %) combined survey and observational data [115].

#### Measures

Studies used the MBI [59] most frequently to assess psychological well-being (14 studies/56 %) [13, 22, 97, 102–106, 108, 111, 112, 114, 116, 117]. Studies measured patient safety using a variety of self-report measures (18 studies/72 %) [14, 22, 23, 97–103, 105, 107, 110–114], with 5 studies (24 %) using objective data such as mortality rates [13, 104, 106, 109, 117]. Two studies (8 %) assessed patient safety via observational data [115, 116].

#### Findings

Authors of the 25 studies examining clinician well-being and patient safety followed two lines of reasoning: Some assumed that committing an error (equaling reduced patient safety) induces (short-term, emotional) distress in clinicians (4 studies/16 %) [13, 97, 100, 103], while the majority of researchers theorized that high (chronic) strain causes employees’ performance to suffer, thus being detrimental to patient safety (20 studies/84 %; box D/E in Fig. 2) [22, 23, 98, 99, 101, 102, 104–113, 115–117]. Overall, results were mixed. Empirical evidence of longitudinal studies lends support to both perspectives [23, 100, 108, 113]. However, due to analytical limitations (i.e., testing for reverse causal relationships), we can draw no definite conclusions [23, 108, 113]. Authors of the 25 studies examining clinician occupational well-being and patient safety reported 123 relationships altogether, of which 64 (52 %) were significant and in line with hypotheses. Of these significant relationships, 42 (66 %) described a positive association between negative indicators of both clinician occupational well-being and patient safety, whereas one (2 %) described a positive association between a positive indicator of clinician occupational well-being and patient safety. Sixteen (25 %) of relationships were negative, describing associations between negative indicators of clinician occupational well-being and positive indicators of patient safety or vice versa. Another five (7 %) associations were unexpected, such as an association between burnout dimension depersonalization and perceived patient safety or heart rate (an indicator of stress) and time spent on cardio-pulmonary resuscitation (an indicator of performance) [102, 109]. However, the latter can be explained by the physically strenuous nature of resuscitation, which is likely to cause an elevated heart rate. Effect sizes ranged from small (OR = 1.09) to large (OR = 8.3, see Table 4).

### Relationships between teamwork, clinician occupational well-being and patient safety

#### Design & sample

Five of the 98 reviewed studies examined teamwork, clinician occupational well-being and patient safety (Table 5), three of which (60 %) sampled nurses only [119, 121, 122]. All studies were cross-sectional self-report studies, with one study (20 %) using risk-adjusted morbidity and mortality rates as objective patient safety indicators.

#### Measures

Three of the studies (60 %) used the nurse-physician-relations scale of the NWI [58] to assess teamwork, and (parts of) the MBI [59] or its emotional exhaustion subscale to measure well-being [119, 120, 122].

#### Findings

Studies examining relationships between teamwork, clinician occupational well-being and teamwork focused exclusively on interpersonal team processes. One study (20 %) proposed a model with the teamwork variable psychological safety [123] serving as a mediator between work environment and work engagement, commitment, and patient safety [120]. However, this mediation effect was statistically non-significant. Another study found a partial mediation between nursing work environment (including nurse-physician relations) and adverse events via burnout. Three studies (60 %) covered teamwork, clinician occupational well-being and patient safety amongst other aspects of the (nursing) work environment, but did not analyze the variables simultaneously, and reported mixed results [118, 121, 122]. Altogether, the five studies reported 33 associations between teamwork, clinician occupational well-being and patient safety, 21 (63 %) of which were significant and in line with authors’ assumptions. These 21 associations included five (23 %) negative associations between teamwork and a negative indicator of patient safety, teamwork and a negative indicator of clinician occupational well-being, and clinician occupational well-being and a negative indicator of patient safety. The 16 positive associations (76 %) included relationships between teamwork and patient safety, clinician occupational well-being and patient safety, and between negative indicators of clinician occupational well-being and negative indicators of patient safety.

Effect sizes ranged from small (*r* = 0.13) to medium (*r* = 0.39).

### Integrative framework

Our aim was to develop a framework applicable to many different healthcare teams in hospital settings. We combined psychological models of team performance and work strain with the findings and theoretical assumptions of this review to formulate specific hypotheses regarding the relationships between teamwork, clinician occupational well-being and patient safety (Fig. 2).

Drawing from the job demands-resources model, we propose that teamwork can be a demand or a resource [29]. This model proposes two parallel processes that influence positive and negative aspects of occupational well-being, such as work engagement and burnout. Job demands deplete the individual’s energy and eventually decrease occupational well-being. Job resources, on the other hand, help employees attain goals, increase occupational well-being or reduce the strain caused by job demands [29].

A team in which actions are not well-coordinated (action team processes), goals are not communicated (transition team processes) and employee’s input to the team is not welcomed by fellow team members (interpersonal team processes) may be demanding for its members and thus directly decrease the team’s ability to provide safe patient care (Fig. 2, arrow C) [10, 11, 25, 120, 123–126]. Simultaneously, ineffective teamwork may lead to decreased clinician occupational well-being: according to the conservation of resources theory, decreased well-being can develop if there is an imbalance between resource investment and resource gain [22, 55, 107, 127]. Ineffective teamwork, as a lack of resource, can lead to a higher individual workload or emotional distress, thereby decreasing well-being [55, 56].

Poor well-being, in turn, may decrease clinicians’ ability to provide safe care (arrow D), because clinicians’ physical and mental resources are depleted [128], cognitive functioning may suffer and they may not be able to exhibit safe working behaviors [129, 130]. The effects of decreased clinician well-being might also be reflected in the team, because distressed team members may not be able to execute relevant team behaviors as effectively (arrow B) [54].

In contrast, if teamwork quality is high, teamwork may act as a resource supporting clinicians to provide safe patient care (e.g., developing shared team mental models, backup behaviors, high psychological safety encouraging clinicians to speak up; or transition, action, and interpersonal team processes; arrow C) [10, 65, 120, 123, 124, 131]. Effective teamwork helps to balance workload, prevent errors, and provide social support in a demanding work environment [126, 132], and may also lead to lower strain levels (arrow A), thereby indirectly supplying clinicians with resources needed for safe patient care (arrow D) [42, 55].

From the reviewed studies, it is not clear whether patient safety influences clinician occupational well-being or vice versa. Clinicians with reduced well-being may not be able to care for patients as safely and effectively due to depletion of resources [23]. Conversely, being involved in an adverse event may lead to guilt and emotional stress potentially compromising psychological well-being in the short- or long-term. [24] Given the existing evidence, we hypothesize that clinician occupational well-being and patient safety are tightly coupled: Tangible patient safety incidents are likely to cause short-term emotional distress [103] and chronic strain in clinicians [24]. Several authors have recognized that, after the patient, the clinician may become the second victim following an adverse event. They may be blamed for errors and have their clinical competence questioned. Sufficient support systems or policies to deal with the effects of error on second victims, such as feelings of anxiety, guilt or shame, do not always exist. [133–135] Chronic strain may also develop due to demanding working conditions which may decrease clinicians’ motivation and efficiency, which could lead to reduced patient safety in the long run (arrows D and E) [23].

### Gaps and trends in current research

One aim of this study was to point out current gaps and recommendations to inform future studies addressing the relationships between teamwork, clinician occupational well-being and patient safety. These gaps and recommendations based on the reviewed studies are summarized in Fig. 2. We found that a holistic approach taking account of the complexity of teams in terms of team structure and different teamwork processes in healthcare organizations was missing, especially in survey studies: for instance, in addition to focusing on the individual professions *within* the team, the entire multi-professional team should be included (e.g., box A/B). Potential multiple team memberships, measures covering transition, action, and interpersonal teamwork processes, and adoption of a temporal rather than static perspective to account for the temporal instability of healthcare teams should be considered (boxes 1 and C in Fig. 2) [136–138]. For example, future studies might employ the team classification developed by Andreatta, which distinguishes between four different team types by classifying team membership and team roles as stable versus variable [139]. Moreover, correlating teamwork behaviors and patient safety indicators over an entire shift is not sufficient to gain an understanding of how they are linked. Instead, changes during the course of a shift or a specific task together with other influencing factors such as disturbances or interruptions need to be taken into account [74, 140].

Future approaches should consider reciprocal relationships between clinician occupational well-being and patient safety, and broaden the assessment of well-being to acute strain, physiological stress indicators or positive outcomes such as work engagement (box 2 in Fig. 2) [141].

With respect to patient safety, there is a clear need to consider how teamwork and well-being interact and impact upon objective safety indicators (boxes D/E and 3 in Fig. 2). This also includes ensuring independence of the objective indicators from other variables. For instance, measuring patient safety via subjective ratings or incident reports may not shed light on a unit’s safety, but rather measure clinicians’ willingness to report errors, which will be higher for clinicians working in a positive team climate [93, 142]. Yet, there seems to be a gap between the need for safety indicators that are feasible and a lack of theoretical discussion of what these indicators actually entail.

We identified several conceptual and methodological issues overarching all three concepts, which could be addressed through more focused study designs (bottom box in Fig. 2). These issues included missing or unclear theoretical foundations, definitions of key concepts, research goals and hypotheses, use of instruments with low validity (despite availability of valid instruments), incomplete description of analyses and reporting of results, mismatch of analyses and research question, and overgeneralization of results.

However, none of the studies suffered from all these drawbacks and many studies investigated the larger work environment so that the comprehensive measurement of teamwork, clinician occupational well-being and patient safety was not within the scope of these studies. Despite the gaps we identified, a large proportion of the reviewed studies were of high methodological quality, using triangulated data, validated instruments and statistical analyses of adequate complexity. Still, validity of results could be greatly improved by supporting pragmatic reasoning with sound theory to define key concepts and formulate clear, measurable research goals and hypotheses. In addition, it will be easier to perform analyses accounting for complexity of both the setting and data (i.e., structural equation or multilevel modeling, longitudinal studies, non-dichotomization of continuous variables).

Altogether, we found the most recent studies seem to address the issues mentioned above, i.e., by employing longitudinal research designs, sampling multi-professional teams or including objective measures of patient safety.

## Discussion

This review provides an overview of the current state of research by scrutinizing relationships between teamwork, clinician occupational well-being and patient safety in hospital settings. Overall, ample evidence on associations between combinations of either two of these concepts exists. The volume and diversity of studies highlight the relevance of these concepts and provide a rich source of information for the design of future studies and interventions. Furthermore, the findings of the review in combination with psychological theories served as the foundation for the framework to explain interrelations between the concepts. The framework is intended to aid interpretation of findings, inconsistencies, and gaps in current research, to serve as a blueprint to designing future studies aiming to improve teamwork, clinician psychological well-being and patient safety.

### Need to explore mechanisms behind relationships

Based on this review, the fact that some studies found no or only partial support for their hypotheses and reported small effect sizes is mainly due to the aforementioned conceptual and methodological issues, rather than non-existent relationships between concepts. These issues could be addressed by utilizing more stringent study designs. For instance, one may not find a relationship between general perceptions of teamwork and objective patient safety indicators. However, a targeted approach that draws from theory on aspects of teamwork and error types and uses validated measures may show that distorted shared mental models are related to inadequate nursing care.

Five of the 98 studies investigated relationships between all three concepts. These five, rather recent and very diverse studies did not provide a sufficient basis for drawing conclusive conclusions regarding the causal mechanisms between the concepts (e.g., because the entire team was not sampled, contradictory results were found across the studies), but demonstrate that the need for an integrative approach has been recognized.

The next step would be to design coherent studies based on strong theoretical foundations to uncover the *mechanisms* underlying the well-established relationships between teamwork, clinician occupational well-being and patient safety. Knowledge of these mechanisms may serve as a basis for designing interventions that integrate all three concepts.

### Adopting an integrative approach

Teamwork is the predominant form of work organization in healthcare. Clinician occupational well-being and patient safety develop in a teamwork context and are dependent on each other. Consequently, clinician occupational well-being and patient safety should not be viewed as outcomes to be managed separately. They may even seem contradictory - additional policies to ensure patient safety may increase clinician workload and decrease well-being. Our findings suggest that they can be integrated into a comprehensive approach: Teamwork may serve as a means to improve both these central organizational outcomes. Also, team-based interventions may be utilized to benefit from the synergies between teamwork, clinician well-being and patient safety. To achieve this, it is essential to focus on multi-professional teamwork and include nurses, physicians and other healthcare professionals. For example, differences in perceptions of teamwork quality by different professions [143, 144] and different approaches to team tasks may result in interpersonal friction [145] and decreased team effectiveness [5, 12]. Aside from proposing general mechanisms between teamwork, clinician well-being and patient safety, the review and framework provide an overview of the specific aspects (i.e., chronic and acute strain, interpersonal, action and transition team processes) that may help target particular problems.

### Outlook

The findings of this review have implications for researchers, and the proposed framework can help to address them in an integrative manner (Fig. 2).

1. *Comprehensive approach to teamwork, well-being and patient safety*
  There is a clear need to investigate teamwork, clinician occupational well-being and patient safety simultaneously in order to evaluate the complex interrelations between these constructs. Interdisciplinary exchange (e.g., medical, nursing, psychological) during study design would help harvest the full potential of studying these associations. Understanding these relationships may help develop interventions aimed at improving all three concepts.
2. *Exploration of causal relationships*
  Little is known about the causal associations between teamwork, clinician occupational well-being and patient safety, and their changes over time. Theoretically informed longitudinal studies and practical interventions will shed more light on this issue. Designing and implementing team-based interventions may investigate the simultaneous effect of improved teamwork on clinician occupational well-being and patient safety.
3. *Considering the entire healthcare team*
  Inter-professional tasks are inherent in healthcare. Thus, only considering nurses *and* physicians (and other healthcare professionals as appropriate) will provide a comprehensive picture of the complex associations between teamwork, clinician occupational well-being and patient safety. In addition, the complexity of teams in healthcare (i.e., temporal instability) needs to be taken in to account [136–139]. In practice, consideration of the entire healthcare team is likely to increase the impact of team-based interventions on clinician and patient outcomes [146].

### Limitations

Although we employed a rigorous search strategy, we may have missed relevant studies. For instance, the lack of consensus between different research approaches concerning terminology for key concepts may have resulted in ambiguous database indexing. However, we compensated for this limitation by including a thorough search of reviews and reference lists. Second, qualitative and interventional studies might have provided additional insights, but – with one exception [88] – were excluded because they did not examine statistical relationships between the concepts that were the focus of this review. Third, study selection, data extraction and rating of study quality were naturally influenced by authors’ reporting style. Nevertheless, the detailed review procedure including structured quality rating proved useful in exploring strengths and weaknesses of the selected studies and thus provided a solid foundation for framework development. Fourth, since disagreements between raters regarding study quality were resolved by consensus discussion, interrater reliability was not calculated. Fifth, we limited this review to acute care hospital contexts, thus, we cannot be sure that our findings are applicable to other (healthcare) settings. However, while other healthcare settings, such as primary care, may differ in terms of team structure or risks to patient safety, we are nevertheless convinced that the overarching issues of this review mentioned in the section above are worth addressing in other contexts. Lastly, as with all reviews, there is always a possibility of publication bias, because non-significant results are often not published.

## Conclusion

We identified substantial relationships between combinations of two of the three concepts teamwork, well-being and patient safety, indicating that all three might influence each other. The proposed framework is based on solid research and provides a foundation for overcoming current research gaps and inconsistencies by hypothesizing causal mechanisms between the concepts and investigating relationships between all three concepts simultaneously. In the most recent studies, we identified a trend to address these gaps. Following the three main recommendations (i.e., comprehensive approach to teamwork, clinician well-being and patient safety; consideration of the entire healthcare team and exploration of causal relationships) will generate research that substantially explores and supports the hypothesized links between teamwork, clinician occupational well-being and patient safety. An integrative perspective of the synergies between teamwork, well-being and patient safety will inform future research, and aims to benefit clinicians and patients alike.

## Abbreviations

AHRQ, agency for healthcare research and quality; AIC, akaike information criterion; ANOVA, analysis of variance; ANTS, anesthetist’s non-technical skills; ATLS, advanced trauma life support; ATOM, anti-air teamwork observation measure; AW, annalena welp; BIC, bayesian information criterion; CBI, Copenhagen burnout inventory; CD-RISC, Connor-Davidson resilience scale; CFI, comparative fit index; CI, confidence interval; CLABSI, central line associated bloodstream infections; CoMeT–E, coordination system for medical teams - emergency; CPR, Cardio-pulmonary resuscitation; CQS, chirurgisches qualitätssiegel survey; GEE, generalized estimating equations; GHQ-12, general health questionnaire; GLM, generalized linear mixed model; icu, intensive care unit; JV, Johanna Vogt (see Acknowledgments); LQWQ-N, Leiden quality of work questionnaire for nurses; M, mean; MBI, Maslach burnout inventory; MD, Mariel Dardel; MeSH, Medical subject heading; MISSCARE, missed nursing care; NNFI, non-normed fit index; NOTECHS, surgical non-technical skills system (observational instrument); NOTSS, non-technical skills for surgeons; NRP, neonatal resuscitation program; NS, not significant; NWI, nursing work index; NWI-R, nursing work index revised; OCHRA, observation clinical human reliability assessment; OLBI, Oldenburg burnout inventory; OR, odds ratio; OTAS, observational teamwork assessment for surgery; RMSEA, root mean square error of approximation; RR, risk ratio; SAQ, safety attitudes questionnaire; SD, standard deviation; SEM, structural equation modeling; SF-36, short form health survey; SOS, safety organizing scale; SS, Sven Schmutz (see Acknowledgments); SSI, standard shiftwork index; STAI, state-trait anxiety inventory; TCI, team climate inventory; TCT, team check-up tool; TEAM, team emergency assessment measure; TeamSTEPPS, team strategies and tools to enhance performance and patient safety; TLI, Tucker Lewis index; TM, Tanja Manser; TPQ, teamwork perceptions questionnaire; UBOS, Utrecht burnout scale; UWES, Utrecht work engagement scale

## Additional files

Additional file 1: Exemplary Search Strategies. (PDF 281 kb) Additional file 2: Quality rating questions. (PDF 196 kb)
