# Supplementary material for: Integrating teamwork, clinician occupational well-being and patient safety – development of a conceptual framework based on a systematic review
Source: BMC Health Serv Res. 2016 Jul 19;16:281. doi: 10.1186/s12913-016-1535-y (PMC4950091; doi:10.1186/s12913-016-1535-y)
Supplement: Additional file 1: — Exemplary Search Strategies. (PDF 281 kb) [file 12913_2016_1535_MOESM1_ESM.pdf]

Online Supplement 1 – Exemplary Search Strategies

Please refer to Methods/Search Strategy in the main manuscript for more information.

Basic search strategy

((teamwork AND patient safety) OR (teamwork AND well-being) OR (patient safety AND well-being))

Exemplary search strategy for PsycInfo database

1. (Team$4 or (leader$4 adj5 team$4)).ti.

2. ((Health adj3 (clinician$1 or physician$1 or nurse$1 or doctor$1 or employee$1 or 'work force' or workforce or staff or surgeon$1 or an?sthesist$1 or worker$1 or personnel)) not 'health care' not 'health system' not 'health reform').ti.

3. (injur$3 adj3 (clinician$1 or physician$1 or nurse$1 or doctor$1 or employee$1 or 'work force' or workforce or staff or surgeon$1 or an?sthesist$1 or worker$1 or personnel)).ti.

4. (Stress$ or Strain$ or Fatigue or 'Well being' or Well-being or Well being or Burnout or 'work Engagement').ti.

5. 1 or 2 or 3

6. (Safety or or Error$1 or Mortalit$3 or Morbidit$3 or 'risk factor' or 'infection risk' or Death or Fatalit$3 or Survival or incident$1 or Complication$1 or Pain$1 or Failure$1 or 'Length of Stay' or (patient$1 adj3 outcome$1) or (patient$1 adj3 Risk$1) or (injur$3 adj3 patient$1)).ti.

7. 1 and 5

8. 1 and 6

9. 5 and 6

10. 7 or 8 or 9

11. ((Team$4 or (leader$4 adj5 team$4)) adj3 (clinician$1 or physician$1 or nurse$1 or doctor$1 or employee$1 or 'work force' or workforce or staff or surgeon$1 or an?sthesist$1 or worker$1 or personnel)).ab.

12. ((Health not 'health care' not 'health system' not 'health reform') adj3 (clinician$1 or physician$1 or nurse$1 or doctor$1 or employee$1 or 'work force' or workforce or staff or surgeon$1 or an?sthesist$1 or worker$1 or personnel)).ab.

13. ((Injur$3 or Stress$ or Strain$ or Fatigue or Well-being or Well being or 'Well being' or Burnout or 'work Engagement') adj3 (clinician$ or physician$ or nurse$ or doctor$ or employee$ or 'work force' or workforce or staff or surgeon$ or an?sthesist$ or worker$or personnel)).ab.

14. 12 or 13

15. ((Safety or injur$3 or Error$1 or Mortalit$3 or Morbidit$3 or Risk$1 or 'risk factor' or 'infection risk' or Death or Fatalit$3 or Survival or incident$1 or Complication$1 or Pain$1 or Failure$1 or 'Length of Stay' or outcome$1) adj3 patient$1).ab.

16. 11 and 14

17. 11 and 15

18. 14 and 15

19. 16 or 17 or 18

20. *work teams/

21. *leadership/ or *leadership qualities/ or *leadership style/ or *transformational leadership/

22. 20 or 21

23. *chronic stress/ or *environmental stress/ or *occupational stress/ or *physiological stress/ or *psychological stress/ or *social stress/ or *stress reactions/

24. *Distress/

25. *Well Being/

26. *occupational health/

27. *occupational safety/

28. *Injuries/

29. 23 or 24 or 25 or 26 or 27 or 28

30. *Treatment Outcomes/

31. *"quality of care"/

32. *Treatment Duration/

33. *Postsurgical Complications/

34. *at risk populations/

35. *Mortality Rate/

36. *Morbidity/

37. *errors/

38. *safety/ or *accident prevention/

39. *Failure/

40. 30 or 31 or 32 or 33 or 34 or 35 or 36 or 37 or 38 or 39

41. 22 and 29

42. 22 and 40

43. 29 and 40

44. 41 or 42 or 43

45. (team or team leadership).id.

46. (patient Safety or Mortalit$3 or Morbidit$3 or infection Risk$1 or Survival or complication$1 or 'Length of Stay').id.

47. (occupational Stress$ or occupational health or occupational safety or Fatigue or Wellbeing or 'Well being' or Burnout or 'work Engagement').id.

48. 45 and 46

49. 45 and 47

50. 46 and 47

51. 48 or 49 or 50

52. (patient$1 or Safety or Error$1 or Mortalit$3 or Morbidit$3 or Risk$1 or Death or Fatalit$3 or Survival or Injur$3 or Incident$1 or Complication$1 or Pain$1 or Failure$1 or 'Length of Stay' or outcome$1 or Injur$3 or Stress$ or Strain$ or Fatigue or 'Well being' or Well being or Burnout or 'work Engagement' or health or Team$4 or leader$4 or clinician$1 or physician$1 or nurse$1 or doctor$1 or employee$1 or 'work force' or workforce or staff or surgeon$1 or an?sthesist$1 or worker$1 or hospital$1 or personnel).ab.

53. (patient$1 or Safety or Error$1 or Mortalit$3 or Morbidit$3 or Risk$1 or Death or Fatalit$3 or Survival or Injur$3 or Incident$1 or Complication$1 or Pain$1 or Failure$1 or 'Length of Stay' or outcome$1 or Injur$3 or Stress$ or Strain$ or Fatigue or 'Well being' or Well being or Burnout or 'work Engagement' or health or Team$4 or leader$4 or clinician$1 or physician$1 or nurse$1 or doctor$1 or employee$1 or 'work force' or workforce or staff or surgeon$1 or an?sthesist$1 or worker$1 or hospital$1 or personnel).ti.

54. (patient$1 or Safety or Error$1 or Mortalit$3 or Morbidit$3 or Risk$1 or Death or Fatalit$3 or Survival or Injur$3 or Incident$1 or Complication$1 or Pain$1 or Failure$1 or 'Length of Stay' or outcome$1 or Injur$3 or Stress$ or Strain$ or Fatigue or 'Well being' or Well being or Burnout or 'work Engagement' or health or Team$4 or leader$4 or clinician$1 or physician$1 or nurse$1 or doctor$1 or employee$1 or 'work force' or workforce or staff or surgeon$1 or an?sthesist$1 or worker$1 or hospital$1 or personnel).ab,ti.

55. 10 and 52

56. 19 and 53

57. 44 and 54

58. 51 and 54

59. 56 or 57 or 58 or 59

60. limit 59 to (human and english language and yr="2000 - 2015")
