# Supplementary material for: Integrating teamwork, clinician occupational well-being and patient safety – development of a conceptual framework based on a systematic review
Source: BMC Health Serv Res. 2016 Jul 19;16:281. doi: 10.1186/s12913-016-1535-y (PMC4950091; doi:10.1186/s12913-016-1535-y)
Supplement: Additional file 2: — Quality rating questions. (PDF 196 kb) [file 12913_2016_1535_MOESM2_ESM.pdf]

Quality rating questions

Adapted from Buckley[1] and Downs and Black[2]

To systematically assess study quality, we combined the systems devised by Buckley and Downs and Black.[1, 2] Ratings were based on a maximum of the 19 items below (not all items were applicable for all studies). Items were rated as 0 = major limitations/not applicable/not mentioned, 0.5 = some limitations, or 1 = fulfilled. Two raters (AW and MD) independently evaluated study quality and resolved disagreements through discussion.

1. Is the theoretical foundation/study background/past research/research gap clearly described?
2. Is the research question(s) or hypothesis clearly stated?
3. Are the main outcomes & predictors to be measured clearly described in the Introduction or Methods section?
4. Are the methods of data collection reliable and valid for the research question and context?
5. Were all relevant ethical issues addressed?
6. Is the subject group appropriate for the study being carried out?
7. Have subjects dropped out? Is the attrition rate less than 50%? For questionnaire based studies, is the response rate acceptable (60% or above)?
8. Was missing data handled appropriately?
9. Was there adequate adjustment for confounding in the analyses from which the main findings were drawn?
10. Are statistical methods/analyses/procedures clearly described?
11. Are the statistical or other methods of results analysis used appropriate (matching research questions, hypotheses, data)?
12. Are the results reported clearly and correctly?

13. Were results supported by data from more than one source?

14. Is it clear that the data justify the conclusions drawn?

15. Could the study be repeated by other researchers?

16. Does the study look forwards in time (prospective) rather than backwards (retrospective)?

17. Are the interventions of interest clearly described? (intervention studies only)

18. Is there a comparison between treatment and control group? (intervention studies only)

19. Was there an additional follow up control measure after the intervention (to investigate long-term effects)? (intervention studies only)

References

1. Buckley S, Coleman J, Davison I, Khan KS, Zamora J, Malick S et al. The educational effects of portfolios on undergraduate student learning: A Best Evidence Medical Education (BEME) systematic review. BEME Guide No. 11. Medical Teacher. 2009;31(4):282–98. doi:doi:10.1080/01421590902889897.

2. Downs SH, Black N. The feasibility of creating a checklist for the assessment of the methodological quality both of randomised and non-randomised studies of health care interventions. Journal of Epidemiology and Community Health. 1998;52(6):377–84. doi:10.1136/jech.52.6.377.
